# Abasic site repair in APE1-deficient mouse embryonic stem cells: a matter for the MRN complex and ATM signaling

**DOI:** 10.1093/nar/gkag921

**Published:** 2026-09-24

**Authors:** Alexandra M Hrovat, Faiza Noreen, Simon D Schwarz, Ezgi M Aksu, Anna Kuśnierczyk, Navnit K Singh, Shana J Sturla, Primo Schär

**Affiliations:** Department of Biomedicine, University of Basel, Basel 4058, Switzerland; Department of Biomedicine, University of Basel, Basel 4058, Switzerland; Swiss Institute of Bioinformatics, Basel 4031, Switzerland; Department of Biomedicine, University of Basel, Basel 4058, Switzerland; Proteomics and Modomics Core Facility (PROMEC), Norwegian University of Science and Technology (NTNU), and the Central Norway Regional Health Authority, Trondheim 7491, Norway; Proteomics and Modomics Core Facility (PROMEC), Norwegian University of Science and Technology (NTNU), and the Central Norway Regional Health Authority, Trondheim 7491, Norway; Department of Health Sciences and Technology, ETH Zurich, Zurich 8092, Switzerland; Department of Health Sciences and Technology, ETH Zurich, Zurich 8092, Switzerland; Department of Biomedicine, University of Basel, Basel 4058, Switzerland

## Abstract

The dual role of base excision repair (BER) in resolving DNA base lesions and enabling active DNA demethylation positions BER proteins as central regulators of both genetic and epigenetic integrity. While BER and active demethylation converge at the generation of AP sites, catalyzed by DNA glycosylases such as thymine DNA glycosylase (TDG), the relative contribution of each pathway to AP site formation and the cellular fate of these lesions remain unresolved. Using APE1-deficient mouse embryonic stem cells (mESCs), with inducible TDG depletion, we reveal that TDG activity accounts for a substantial fraction of AP sites in mESCs, establishing a direct link between active DNA demethylation and APE1-mediated BER. We demonstrate that APE1 is essential for mESC differentiation, underscoring its role in processing AP sites generated during epigenetic reprogramming. Through a genome-wide CRISPR/Cas9 screen designed to map the broader functional interactome of APE1, we identified unexpected genetic dependencies that extend beyond DNA repair. We also found that DNA double-strand break repair factors act outside their canonical pathways, cooperating with nucleotide excision repair and translesion synthesis to compensate for the loss of APE1. These findings highlight novel compensatory DNA end-processing mechanisms that safeguard genomic integrity in the absence of APE1, revealing a high level of plasticity of DNA repair in mESCs.

## Graphical Abstract

**Graphical Abstract:**
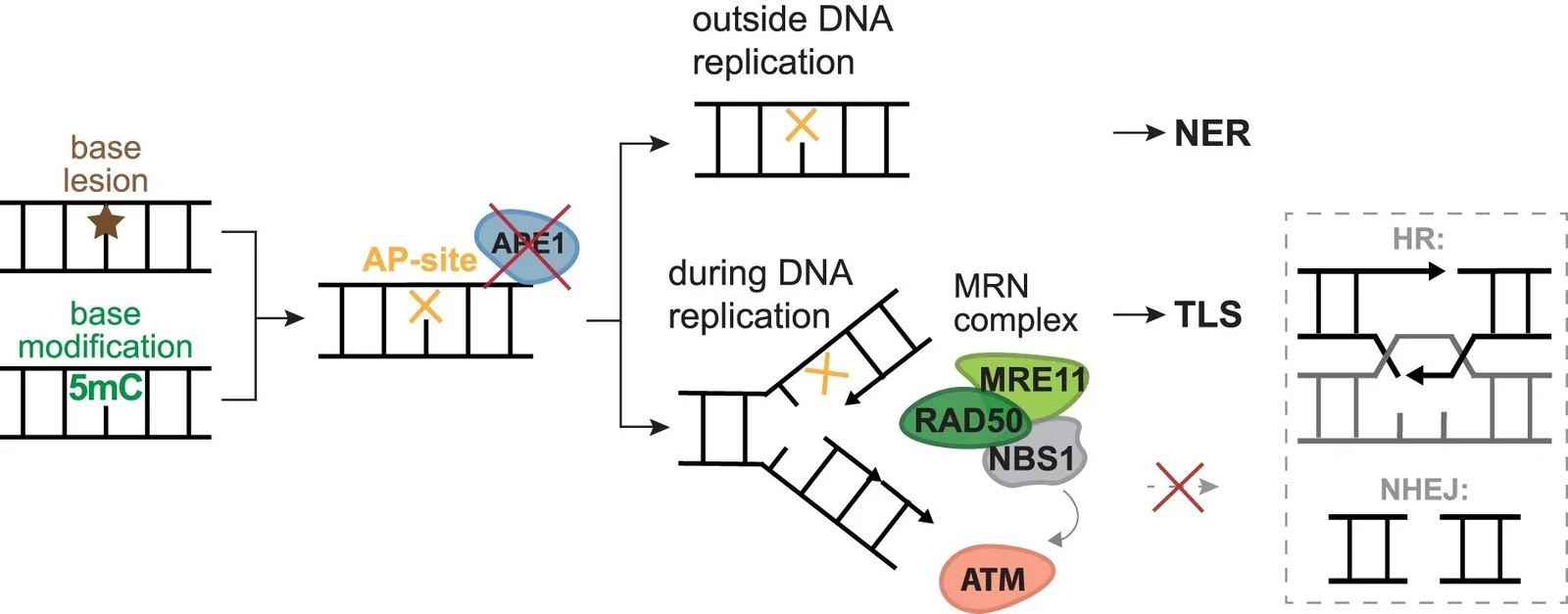

## Introduction

DNA damage is a challenge for all living cells, arising from both, endogenous metabolic activities and exogenous environmental impacts [1]. To maintain genomic integrity, cells rely on a complex DNA damage response (DDR) network that coordinates the detection, signaling, and repair of the variety of DNA lesions. This network consists of different DNA repair systems tailored to address specific types of DNA damage: homologous recombination (HR) and non-homologous end joining (NHEJ) are the main pathways for repairing DNA double-strand breaks (DSBs), nucleotide excision repair (NER) eliminates bulky, helix-distorting lesions, and base excision repair (BER) addresses small lesions caused by base oxidation, alkylation, or deamination [2].

Among these pathways, BER stands out for its dual purpose, as it not only processes mutagenic or cytotoxic DNA base lesions but also replaces physiologically modified bases, such as the oxidative derivatives of 5-mehtylcytosine (5mC) generated by ten-eleven translocation (TET) enzymes, which have genome regulatory functions [3, 4]. Conventional BER, or damage-triggered BER, is initiated by a variety of DNA glycosylases that excise specific types of base lesions. Monofunctional glycosylases generate apurinic/apyrimidinic sites (AP sites), which are then processed by the AP endonuclease APE1 (also known as REF-1) to DNA single-strand breaks (SSBs). By contrast, bifunctional DNA glycosylases possess AP lyase activity and directly incise AP sites. Yet, BER initiated by bifunctional DNA glycosylases still requires further SSB 3′- end processing for repair completion, a step that may also involve APE1 [5–7]. These resulting SSBs are then repaired by a coordinated mechanism involving poly [ADP-ribose] polymerase 1 (PARP1), X-ray repair cross-complementing protein 1 (XRCC1), DNA polymerase β (POLβ), and DNA ligase III (LIGIII) [8]. BER targeting the 5mC oxidation products, 5-formylcytosine (5fC) and 5-carboxylcytosine (5caC) initiates by engagement of one specific DNA glycosylase, the thymine DNA glycosylase (TDG), generating an AP site, which is then further processed along the repair steps described for conventional BER to insert an unmodified cytosine [9, 10]. Unlike other DNA glycosylases, TDG disruption does not increase mutation rates but causes aberrations in epigenetic DNA and histone modifications, as well as gene expression in cells undergoing differentiation, making it indispensable for mouse embryogenesis. This phenotype highlights the unique role of TDG-initiated BER in DNA methylation control and its redundancy in conventional BER [11–13].

APE1 lies at the intersection of all BER pathways, acting on AP sites regardless of their origin. So far, only one report in mouse zygotes provides experimental evidence for a role for APE1 in DNA demethylation. In this study, the inhibition of the endonuclease function of APE1 caused an increased 5mC staining in the paternal pronucleus, which was interpreted as a blockage of DNA demethylation [14]. However, whether TET and TDG, and hence BER, play a role at this early developmental stage, when global 5mC erasure is observed in the paternal nucleus, remains uncertain [15]. Nevertheless, the importance of APE1 in embryonic development is underlined by developmental failure of mice [16], zebrafish [17], and Xenopus [18] carrying APE1 defects. Moreover, full APE1 depletion has been reported to be cell-lethal in various cell types [19, 20], whereas some cell models appear to tolerate APE1 loss, including HEK293FT human embryonic kidney cells [21], HCC1937 breast cancer cells [22], CH12F3 mouse B cells [23], mouse embryonic stem cells (mESCs) [18], and esophageal squamous cell carcinoma (TE-1) [24]. These divergent observations may indicate the existence of alternative pathways compensating for APE1 loss with varying efficiency across different cell lines. Indeed, several enzymes with AP site incision or SSB end-processing function exist and have been proposed to act as backup for APE1 deficiency [25–28]. Regarding BER as a whole, other DNA repair pathways have been proposed and shown to act as backup, including HR and NER [29–31]. While these observations provide important hints toward alternative AP site repair pathways, APE1 has also been associated with functions other than DNA repair [32], e.g. transcriptional regulation [33] and RNA metabolism [34], but the overall cellular consequences to its loss have not been systematically addressed.

To investigate the spectrum of biological functions of APE1, we examined the phenotypic consequences of *Ape1* deletion and identified proteins and pathways that compensate for its loss. To assess APE1 function in both canonical and 5mC oxidation-associated BER, we used mESCs, where TET-TDG-mediated DNA demethylation activity is comparably high [35, 36]. We thus generated APE1 knockout clones in the background of a conditional *Tdg* knockout system [37] to be able to control AP site generation by 5fC and 5caC excision. Following phenotypic characterization of APE1 loss, we used this model to perform an unbiased genome-wide CRISPR screen for gain and loss of fitness factors in the background of an APE1 deficiency. Notably, we found that TDG depletion rescues specific phenotypes observed in APE1-deficient mESCs, establishing APE1 as the primary endonuclease to process AP sites generated during active DNA demethylation. CRISPR screening identified multiple functional APE1 interactions with and beyond DDR and repair pathways. Curiously, signatures of identified compensatory DDR pathways varied between different APE1-deficient mESC clones, implicating the existence of different adaptation mechanisms upon *Ape1* deletion.

## Materials and methods

### Cell culture and differentiation

mESCs were generally cultured at 37°C in a humidified incubator with 5% CO_2_ in serum-free 2i medium supplemented with 1000 U/ml leukemia inhibitory factor (LIF; Polygene, 10015303). TDG knockout induction was performed as described earlier [37]. In short, tamoxifen (4-OHT, Sigma–Aldrich H7904) was dissolved in dimethyl sulfoxide (DMSO; Sigma–Aldrich D2650) and applied at 3 µM or mock-treated with DMSO for 2 h before the medium was replaced. TDG depletion was verified by immunoblotting.

Neuronal differentiation was carried out as previously described [36, 38]. In summary, mESCs were cultured for 16 h in ESC medium [ESCM; Dulbecco’s modified Eagle’s medium (DMEM) high glucose (Sigma–Aldrich, D5796), 15% heat-inactivated fetal bovine serum (FBS; PAN Biotech, P30-3031) with LIF], before seeded onto non-adherent bacterial dishes (Greiner Bio-One) in ESCM without LIF for embryoid body (EB) formation. After 4 days and daily ESCM exchange, 5 µM all-trans retinoic acid (RA; Sigma–Aldrich, R2625) was added, and incubation continued for another 4 days. EBs were dissociated, filtered, and plated onto poly-L-lysine (Sigma–Aldrich, P6282) and laminin (Sigma–Aldrich, L2020)-coated dishes in N2 medium [DMEM-F12 (Thermo Fisher, #21331020), 1× N2 supplement (Thermo Fisher, #17502048)]. N2 medium was exchanged after 2 h, 24 h, and after 48 h and then replaced with B27 medium [Neurobasal medium (Thermo Fisher, #21103049), 1× B27 supplement (Thermo Fisher, #12587010)].

### Generation of APE1 knockout mESCs

APE1 knockout mESCs were generated using CRISPR/Cas9-mediated genome editing. Two guide RNAs (gRNAs) targeting *Ape1* locus were designed, with the following sequences: gRNA1: AAAACCGAGAAGGAGGCCGC; gRNA2: CGACGGGGAAGAACCCAAGT. Efficiency and specificity were assessed according to UCSC genome browser [39] (Moreno Mateos T7 score raw). For transfection, ribonucleoprotein (RNP) complexes were assembled following the IDT RNP complex protocol, with Transit-X2 (Mirus Bio Corporation; MIR 6003) used as the transfection agent and a GFP-expressing plasmid included as a co-transfectant. Briefly, the RNP complex was incubated with Transit-X2 and the GFP plasmid for 15 min at room temperature (RT) before being added to singularized mESCs and incubated at 37°C. Two days post-transfection, GFP-positive cells were sorted by fluorescence-activated cell sorting (FACS) using FACS Aria II system, and single cells were plated into 96-well plates for clonal expansion containing 2i medium. For the first 5–7 days, 2% heat-inactivated FBS (PAN Biotech, P30-3031) was added to the 2i. Parental mESCs were used as wild-type (wt) controls, and no scrambled gRNA control was included.

### Cell doubling time analysis

A total of 0.5 million mESCs were seeded into a 6 cm plate and cultured in 2i medium. After 2 days, cells were harvested, counted using trypan blue exclusion with automated cell counter (Countess), and reseeded at the same density. This process was repeated three times in triplicates. The doubling time over 24 h was calculated based on the cell counts obtained from each cycle.

### Cell cycle analysis

mESCs were cultured in 2i medium, harvested, and washed twice with ice-cold phosphate buffered saline (PBS). Roughly 0.5 million cells were used for each sample. mESCs were fixed by adding ice-cold 70% ethanol dropwise while vortexing to prevent clumping, followed by incubation on ice for 30 min. Next, mESCs were washed twice with PBS before staining with DAPI (1 µg/ml; Invitrogen, D1306) for 10 min at RT. After two additional washing steps, mESCs were resuspended in PBS for flow cytometry analysis. Cell cycle distribution was determined using a CytoFLEX S flow cytometer, applying gating to exclude debris and cell doublets. Analysis was performed with FlowJo software (Version 10) using the inbuilt cell cycle tool.

### Whole cell extracts and western blot

mESCs were cultured in 2i medium, and 50 µM camptothecin (CPT; Sigma–Aldrich, C9911) was added for 24 h as positive control where indicated. To obtain whole cell extracts, cells were harvested and resuspended in NP-40 lysis buffer [20 mM Tris–HCl, 1 mM CaCl_2_, 0.5 mM ethylenediaminetetraacetic acid (EDTA), 10% Glycerol, 2.7 mM KCl, 1 mM MgCl_2_, 125 mM NaCl, 1% NP-40, 1 mM phenylmethylsulfonylfluorid (PMSF), 1 mM dithiothreitol (DTT), 1× PhosSTOP (Roche), 1× Protease inhibitor Complete (Roche)] before boiled for 10 min at 95°C and sonicated for 10 min (30 s ON, 60 s OFF, Diagenode Bioruptor). After separation by glycine-based sodium dodecyl sulfate–polyacrylamide gel electrophoresis (SDS-PAGE), samples were blotted onto a 0.2 μm nitrocellulose membrane (GE Healthcare) for 2.5 h at 300 mA at 4°C in a transfer buffer [25 mM Tris-base, 193 mM glycine, 10% methanol]. The membrane was blocked with 10% milk in TBS-T [10 mM Tris, pH 7.5, 150 mM NaCl, 0.1% Tween-20] for 1 h and then incubated with following antibodies: rabbit polyclonal anti-APE1 antibody (Thermo Fisher, PA5-29157; 1:1000), rabbit polyclonal anti-APE2 antibody (Thermo Fisher, PA5-72607; 1:1000), mouse anti-Tubulin (Sigma–Aldrich, T9026; 1:10 000), mouse anti-Actinβ (Sigma–Aldrich, A5441; 1:10 000), rabbit anti-phospho-p53 (Ser15; Cell Signaling, 9284; 1:1000), mouse monoclonal anti-Cas9 (GeneTex; GTX53807; 1:1000), mouse anti-TDG (4A11, Schär lab; 1:500), diluted in 5% milk for 1 h at RT or overnight at 4°C. After three consecutive washing steps using TBS-T, the appropriate horseradish peroxidase-linked anti-mouse or anti-rabbit IgG secondary antibody (GE Healthcare) was applied, diluted 1:10 000 in 5% milk TBS-T for 1 h at RT. The three washing steps in TBS-T were repeated, and proteins were visualized with WesternBright ECL (Advansta) and detected with a Fusion FX imaging system (Vilber).

### Genomic DNA extraction

For standard isolation of DNA from mESCs, we used the Quick-DNA Miniprep Plus Kit (Zymo; D4069) according to the manufacturer’s protocol. Concentration and quality were measured using DeNovix DS-11 FX + spectrophotometer (DeNovix Inc., USA). To characterize the *Ape1* locus upon CRISPR-Cas mediated disruption, the following primer pairs were used (5′–3′): forward, GTCTCTGGCTTCGTTGGGAG; reverse, ACGAGTCAACCAAGAAAGGTC.

### Gene expression analysis by RT-qPCR

Total RNA was extracted using RNeasy Mini Kit (Qiagen; 74136), with an additional on-column DNaseI (Roche) digestion step. RNA concentration and quality were assessed using DeNovix DS-11 FX + spectrophotometer (DeNovix Inc., USA) and Fragment Analyzer 5200 (Agilent, Waldbronn, Germany), respectively. Complementary DNA (cDNA) synthesis was performed using the RevertAid First Strand cDNA Synthesis Kit (Thermo Fisher, K1691) with oligo-dT primers. Quantitative polymerase chain reaction (qPCR) was carried out with the SensiFAST SYBR No-ROX Kit (Meridian Bioscience, BIO-98005). *Ape1* mRNA was amplified using the following primer pair (5′–3′): forward, GACGGGGAAGAACCCAAGTC; reverse, GTGCTTCTTCCTTTACCCAATCC.

### Ribo-depleted RNA sequencing

Total RNA was extracted as described earlier. RNA library preparation was carried out at the genomics facility Basel, following Illumina Tru-Seq Stranded Total RNA Library Kit with RiboZero depletion. Libraries were sequenced using an Illumina NovaSeq platform in a paired-end 100 bp (PE100) format, generating ~40 million reads per sample. Sequencing was performed in technical triplicates. Reads were aligned to the mouse genome GRC39 (vM33) obtained from GENCODE.

RNA-seq libraries were prepared using the Illumina TruSeq Stranded Total RNA Ribo-Zero, which includes ribosomal RNA depletion. Paired-end strand-specific sequencing was performed. Sequencing reads were quantified using STAR v2.7.9a against the GRCm39 mouse transcriptome. Transcripts with fewer than 15 counts, after normalization for library depth, were excluded from further analysis. Raw counts were normalized using the TMM (Trimmed Mean of M-values) method implemented in the edgeR package [40] to obtain normalized read counts. Voom transformation was then applied to model the mean-variance relationship in the data before performing differential gene expression analysis using the limma package [41] in R (version 4.4.2). Genes were considered significantly up- or downregulated based on a fold change of ≥2 and a false discovery rate (FDR)-adjusted *P*-value of < .05.

### Immunofluorescence staining

mESCs were cultured in 2i medium and seeded on gelatin-coated coverslips. The following day, mESCs were washed with PBS containing 0.1% Tween-20 (PBS-T). For phospho-ATM (p-ATM) staining, mESCs were pre-extracted with CSK buffer [25 mM HEPES, pH 7.4, 50 mM NaCl, 3 mM MgCl_2_, 300 mM sucrose, 0.5% Triton X-100] for 10 min prior to fixation. All cells were then fixed in 2% formaldehyde and blocked in PBS-T containing 5% bovine serum albumin (BSA). Coverslips were incubated with primary antibodies diluted 1:500 in 5% BSA/PBS-T for 1 h: rabbit polyclonal anti-APE1 antibody (Thermo Fisher, PA5-29157), rabbit polyclonal anti-53BP1 (Santa Cruz, sc-22760), mouse monoclonal anti-RAD51 (Novus, 14B4), and mouse monoclonal anti-p-ATM (Ser1981; Cell Signaling, 4526S). Following washing in PBS-T, mESCs were incubated with the appropriate secondary antibodies (Alexa 488- or 594-conjugated anti-mouse/anti-rabbit; Invitrogen), diluted in 5% BSA/PBS-T. mESCs were counterstained with DAPI (1 mg/ml) and mounted using Vectashield mounting medium (Vector Laboratories, H1000). Imaging was performed using a Leica SP5 confocal microscope (Leica DMI 6000 B inverted microscope) for analysis of 53BP1 and RAD51, using a 63× HCX Plan-Apo CS objective, the Leica LAS AF software, and the Leica EL6000 as fluorescent light source. Alternatively, a Leica Stellaris 8 Falcon confocal microscope (fully motorized DMi8 inverted microscope) was used for analysis of APE1 and p-ATM, using a 63× HC Plan-Apo CS2 objective, the Leica LAS X software, and the Leica LED3 as a fluorescent light source. Foci detection was performed with ImageJ/Fiji [42] and the QuaSI-image analysis macro, working on Z-projections (manuscript in preparation, software available at https://github.com/BauerCU/QuaSI).

### HPLC-MS/MS analysis of DNA modifications

To extract high-integrity and large quantities of DNA, the Genomic-tips from Qiagen were used (100/G). *HPLC-MS/MS* analysis was performed by the Proteomics and Modomics Core Facility (PROMEC), Norwegian University of Science and Technology (NTNU).

DNA was enzymatically hydrolysed to deoxyribonucleosides with Benzonase from *Escherichia coli* (*E. coli*; Santa Cruz Biotech), Nuclease P1 from *P. citrinum* (Sigma), and alkaline phosphatase from *E. coli* (Sigma) in 10 mM ammonium acetate buffer pH 6.0, 1 mM magnesium chloride, at 40°C for 40–60 min. Samples were precipitated by addition of three volume equivalents of ice-cold acetonitrile and centrifugation at 16 000 rcf for 30 min at 4°C. The supernatants were collected, freeze-dried, and redissolved in 50 ul of stable-isotope-labeled internal standards (I.S.) for analysis. The chromatographic separation of deoxynucleosides was performed using an Agilent 1290 Infinity II UHPLC system with an ZORBAX RRHD Eclipse Plus C18 150 × 2.1 mm ID (1.8 μm) column protected with an ZORBAX RRHD Eclipse Plus C18 5 × 2.1 mm ID (1.8 µm) guard column (Agilent). The mobile phase consisted of A (water, 0.1% formic acid) and B (methanol, 0.1% formic acid) starting at 0.15 ml/min flow of 5% B for 3 min, followed by 3.5 min gradient of 5%–20% B at flow 0.2 ml/min, 2 min of 20%–95% B, 1 min of 95% B, and 4 min re-equilibration with 5% B at flow 0.23 ml/min. Mass spectrometric detection was performed using an Agilent 6495 Triple Quadrupole system operating in positive electrospray ionization mode. The following mass transitions were monitored: 252.1/136.1 (2′-deoxyadenosine), 228.1/112.1 (2′-deoxycytidine), 268.1/152.1 (2′-deoxyguanosine), 243.1/127.1 (2′-deoxythymidine), 284.1/168.1 (8-oxo-2′-deoxyguanosine), 258.1/142.1 (5-hydroxymethyl-2′-deoxycytidine), 258.1/124.1 (5-hydroxymethyl-2′-deoxycytidine qualifier), 272.1/156.1 (5-carboxyl-2′-deoxycytidine), 259.1/143.1 (5-hydroxymethyl-2′-deoxyuridine), 256.1/140.1 (5- formyl-2′-deoxycytidine), 257.1/136.1 (13C5-2′-deoxyadenosine I.S.), 246.1/130.1 (13C, 15N2-2′-deoxythymidine I.S.), 289.1/173.1 (15N313C2-8-oxo-2′-deoxyguanosine I.S.), 261.1/145.1 (d3-5-hydroxymethyl-2′-deoxycytidine I.S.), 264.1/112.1 (gemcitabine, a dC analog coeluting with 5ca(dC) and used as I.S. for 5f(dC), 5ca(dC), and 5hm(dU)).

### Alkaline comet assay

Microscopy slides (75 × 25 mm) were precoated with 1% normal melting point agarose (Bioline, BIO-41025), followed by a layer of 0.75% low-melting-point agarose (LMPA; SeaPlaque GTG Agarose, Lonza, #50111) in 1× PBS, and stored on ice for 20 min. mESCs were harvested, followed by resuspension in 1× PBS (3 × 10^6^ cells/ml), and mixed with LMPA. Subsequently, 50 µl LMPA-cell mix was spread onto precoated slides and solidified on ice, before covered with a final layer of LMPA. Slides were lysed in Trevigen lysis buffer (R&D Systems, #4250-050-01; 45 min, 4°C, dark), treated with freshly prepared alkaline buffer [200 mM NaOH/1 mM EDTA; 30 min], and subjected to electrophoresis (21 V, 20 min, 4°C). After neutralization and ethanol dehydration, slides were dried overnight, before rehydration and staining with SYBR gold (Invitrogen, S11494) the following day. Imaging of 500 cells per slide was performed using fully motorized Zeiss Axio Imager Z2 scanning microscope (objectives: 20× Plan-Apo, fluorescent light source: HXP, filtercubes: FITC) with Metafer4 software using the Comet Assay customization, excluding manually doublets and apoptotic cells.

### Aldehyde reactive probe assay

mESCs were cultured in 2i medium and treated with or without methyl methanesulfonate (MMS; 1 mM, 1 h; Sigma–Aldrich, 129925). Cells were harvested, and genomic DNA was isolated using the Quick-DNA Miniprep Plus Kit (Zymo; D4069) according to the manufacturer’s instructions. DNA concentration was determined and adjusted using a fluorescence-based assay [DeNovix double-stranded DNA (dsDNA) high sensitivity; TN145]. AP site derivatization with aldehyde reactive probe (ARP) and subsequent detection were performed using the DNA damage detection kit (Dojindo, DK02-12) according to the manufacturer’s protocol.

### Click-fluoro-quant

AP sites were quantified by click-fluoro-quant as described [43]. Genomic DNA (gDNA) was adjusted to 200 ng µl^−1^, and 3 µg (15 µl) per reaction was allowed to react either without enzyme, with 1 U of T4 PNK (NEB, M0236L), or with 1 U of ENDOIV (NEB, M0304L) in 1 × NEBuffer 2 (NEB, B7002S) in a final volume of 20 µl. The reaction mixture was incubated for 30 min at 37°C, immediately followed by the addition of 5 µl of a master mix containing 1 × NEBuffer 2, 250 µM prop-dGTP (Jena Bioscience, custom synthesis), and 2 U of Therminator IX (NEB, custom order). After 10 min of incubation at 60°C, gDNA samples were purified using ProNex magnetic beads (Promega, NG2001) at a beads:sample ratio of 8:5 and eluted in 50 µl of buffer EB (Qiagen, 19086). For CuAAC, the sample volume was condensed to 6 µl using a vacuum concentrator. The reaction was performed with 4 µl of AF594 picolyl azide (Jena Bioscience, CLK-1296-AZ-1; 0.5 mM), 4 µl of DMSO (Sigma–Aldrich, D8418), 1 µl of CuSO_4_ (Sigma–Aldrich, C1297; 20 mM), and 1 µl of THPTA (Lumiprobe, F4050; 200 mM) premixed before addition, and 2 µl of sodium phosphate buffer (Sigma–Aldrich, 71 643 and 71 505; 1 M, pH 7.0). The reaction was started by adding 2 µl of freshly prepared sodium ascorbate (Sigma–Aldrich, 1613509; 400 mM in H_2_O) and incubated for 30 min at 37°C. The reaction product was purified using the Monarch gDNA purification kit (NEB, T3010L) and eluted in 100 µl of buffer EB. gDNA concentration was measured using the Quantus fluorometer with QuantiFluor ONE dsDNA dye (Promega, E4870). For fluorescence analysis, identical amounts of each gDNA sample were mixed with PBS to a final volume of 610 µl. The samples were split into 3 × 200 µl technical replicates (for fluorescence measurement) and pipetted in a nonclear 96-well black plate. For blank measurement, 3 × 200 µl of PBS was added to the plate. Fluorescence was recorded with an Infinite M200 Pro plate reader (Tecan) using the following settings: λEx = 580 nm, λEm = 623 nm, gain = optimal, 25 flashes, 20 µs of integration time, and 25°C. The same gain value was used for replicates of the same experiment. Data were analyzed by subtracting the mean blank fluorescence from all sample fluorescence values. They were further normalized to the DNA amount by dividing each value by the amount of gDNA in the sample in ng. Three technical replicates per sample were averaged. Three click-fluoro-quant experiments were performed, each containing seven samples, and, within each experiment, for each sample, the level of deoxyguanosine-derived AP sites was computed as the difference of normalized fluorescence signal between the ENDOIV and T4 PNK reactions.

### Pulsed-field gel electrophoresis

mESCs were cultured in 2i medium with or without Zeocin (20 µg/ml, 24 h; Gibco, R25001), harvested, and rinsed once in ice cold 1× PBS. For one plug, 0.25 × 10^6^ mESCs were resuspended in 50 µl PBS and mixed with 50 µl of pre-melted low melting agarose (1.5%, NuSieve GTG agarose, Lonza, #50 081) and transferred to a plug mold (BioRad). After solidification at 4°C, the plugs were ejected from the mold and incubated at 37°C for 72 h in lysis buffer [100 mM EDTA, pH 8.0, 0.2% sodium deoxycholate, 1% sarcosine, 1 mg/ml Proteinase K (Roche)]. After lysis, plugs were washed [20 mM Tris–HCl pH 8.0 and 50 mM EDTA], and placed directly onto the electrophoresis comb before 0.9% agarose gel was poured. Gel electrophoresis was performed for 21 h using the CHEF-DR III system (BioRad) at 14°C in 0.5× Tris-Borat-EDTA (TBE) buffer.

### Genome-wide CRISPR knockout screen and data analysis

Cas9-expressing mESC lines were generated by transduction with lentivirus expressing Cas9-GFP (Addgene, #44719). Cas9 activity was validated using a flow cytometry-based assay, in which mESCs were transduced with either a GFP-targeting gRNA or a scrambled control, followed by assessment of GFP signal via flow cytometry. The Mouse Brie CRISPR knockout pooled library (Addgene #73633-LV) [39] was used, which targets the mouse genome with 78 637 gRNAs. mESCs were infected with the lentiviral Brie library at a multiplicity of infection of 0.3 to ensure single gRNA integration per cell. After infection, mESCs were selected with puromycin (Sigma–Aldrich; P8833) for two days to remove non-transduced cells. Following a 1-day recovery period, half of the cells were collected as the initial time point (*T*_0_), corresponding to a coverage of 500 cells per gRNA for APE1 knockout mESCs and 100 cells per gRNA for wt mESCs. The remaining mESCs were cultured for 12 days in 2i medium, after which they were collected as final time point (*T*_n_), at a coverage of 500 cells per gRNA for both cell lines. Genomic DNA was extracted using Wizard Genomic DNA extraction kit (Promega, A1120), and gRNA sequences were amplified and prepared for Illumina sequencing in a one-step polymerase chain reaction (PCR) using barcoded primers and 2× NEBNext Ultra II Q5 Master Mix (NEB, M0544L). The library was sequenced using NovaSeq 6000 system with SP 100 flow cell and single-end reads.

Sequencing reads from the CRISPR screen were aligned to the Mouse Brie CRISPR knockout pooled library (Addgene #73633-LV) using Bowtie 2 [44]. Prior to alignment, reads were trimmed to isolate the gRNA sequence region. The Mouse Brie CRISPR Knockout Pooled Library was indexed using Bowtie 2, and reads were mapped using end-to-end alignment mode with high-sensitivity settings, allowing for minimal mismatches. Successfully aligned reads were quantified by counting the number of reads mapping to each gRNA. Further analysis was performed at the gene level by aggregating gRNA counts targeting the same gene. Raw count data were processed using the *edgeR* package in R [40]. Features with low abundance were filtered by retaining genes with counts per million > 0.5 in at least three samples. Samples with fewer than 100 000 total counts were excluded. The filtered dataset was normalized using the TMM method to adjust for differences in library sizes and composition bias. A design matrix was constructed to model the experimental conditions, and dispersion estimates were calculated to account for biological variability. Gene-wise negative binomial generalized linear models were fitted to the normalized count data, followed by likelihood ratio tests to compare key contrasts: the final time point (*T*_n_) versus the initial time point (*T*_0_) in both wt and APE1 knockout (APE1_null2_) conditions. Significantly enriched or depleted genes were identified based on fold change of ≥2 and a FDR-adjusted *P*-value of < .05.

### Metabolic activity assay

mESC viability was measured using the WST-8-based Cell Counting Kit-8 (CCK-8, MedChem Express, HY-K0301-6X) according to the manufacturer’s instructions. mESCs were seeded in a 96-well plate at a density of 1 × 10^4^ cells per well in 100 µl of 2i medium. The next day, mESCs were treated with the following inhibitors diluted in fresh 2i medium for 24 h: CPT, menadione (Sigma–Aldrich), MMS (Sigma–Aldrich, 129 925), APE1 endonuclease inhibitor (MedChemExpress, CRT0044876), MRE11 inhibitor (mirin; Selleckchem, S8096), ATM inhibitor (Selleckchem, KU-55933), ATR inhibitor (Selleckchem, VE-822), CHK1 inhibitor (Focus Biomolecules, rabusertib), translesion synthesis (TLS) inhibitor (MedChemExpress, JH-RE-06), and NER inhibitor (Sigma–Aldrich, NSC130813). Then, 10 µl of CCK-8 solution was added directly to each well, and the plate was incubated for an additional 2 h at 37°C. The absorbance was measured at 450 nm using a microplate reader. Blank wells containing only medium and CCK-8 without cells were included for background correction. mESC viability was calculated relative to untreated control cells. All experiments were performed in triplicates.

### Sister chromatid exchange assay

Labeling of the DNA of mESCs was performed by culturing mESCs in 2i medium supplemented with 10 µM BrdU (Sigma–Aldrich, B9285) for 48 h. Colcemid (Sigma–Aldrich, #10295892001) was added 1.25 h prior to harvesting. mESCs were collected, washed, and incubated in hypotonic solution for 20 min at 37 °C. mESCs were then fixed in ice-cold methanol:acetic acid (3:1) and washed three times with it, before resuspending in 0.5 ml fixative (for 1 million mESCs). Three drops were applied per slide for metaphase chromosome spreads. Slides were incubated for 72 h at 37°C. Sister chromatid differential staining was achieved by first labeling chromatin with Hoechst (Thermo Fisher, 33258; 15 min), followed by UV-B light exposure (5 J/cm^2^; 1 min), and subsequent Giemsa staining (Sigma–Aldrich, #1092040100; 5 min). Chromosome spreads were analyzed with a widefield fully motorized Zeiss Axio Scanning Microscope, using a scanning stage with eight slides, AxioCam, Zen Blue software, and 63× Plan-Apo objective.

### Competitive growth assay

Cas9-expressing GFP-positive mESCs were mixed with wt GFP-negative mESCs (no Cas9) to control for growth differences. mESCs were then infected with mCherry-labeled lentivirus delivering gRNAs targeting the gene of interest or a scrambled control. Following infection, four distinct populations were measured by flowcytometry (FACS Aria II system): green (non-infected Cas9 mESCs), green and red double-positive (infected Cas9 mESCs), red (infected wt mESCs), and double-negative non-infected wt mESCs. At 3 days post-infection and every second day thereafter, the relative abundance of these populations was assessed via flow cytometry to track the abundance of double-positive mESCs (GFP/mCherry) over time. Cell numbers were normalized to day 3 as initial time point, and also to the simultaneously infected scrambled control population.

## Results

### APE1 is critical for DNA integrity but dispensable for cell viability in naïve pluripotent mESCs

**Figure 1.**
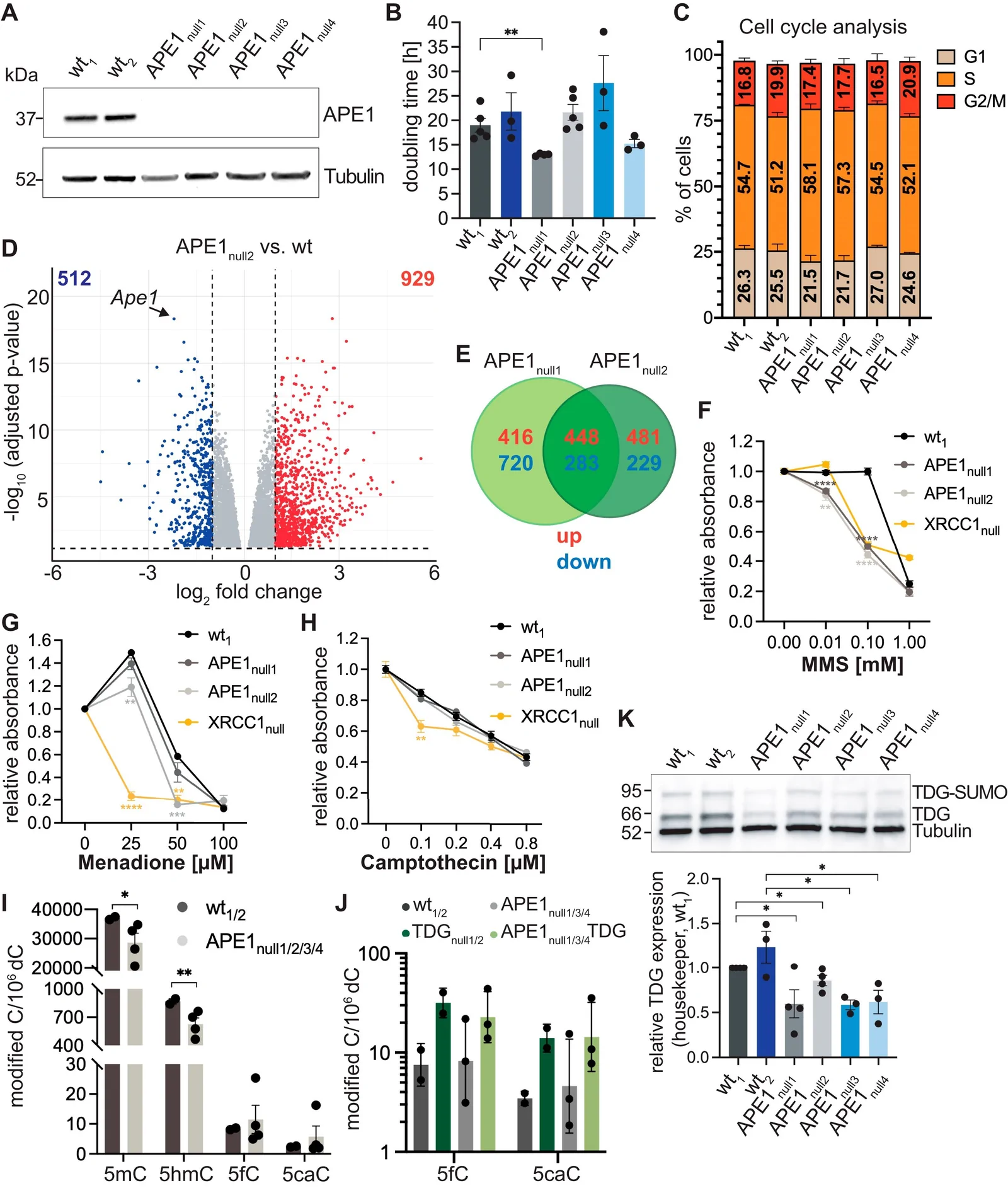
*Ape1* deletion does not impair mESC viability in unchallenged conditions but sensitizes cells to induced alkylating or oxidative DNA damage and perturbs 5mC oxidation. (**A**) Immunoblot analysis for APE1, with Tubulin as loading control, of whole-cell extracts of mESC lines indicated. (**B**) Doubling time of mESC lines, calculated based on the fold increase of cell numbers observed after 48 h of mESC culture in 2i medium. Shown are means with standard errors of three independent experiments. (**C**) Flow cytometric analysis of cell cycle stage (G1, S, G2/M phase) distribution of mESC lines indicated using DAPI staining. Shown are means with standard errors of three independent experiments. (**D**) Ribo-depleted RNA-seq showing differential gene expression between APE1_null1_ and wt_1_ mESCs. Significantly (log_2_FC > ±1, FDR < 0.05) downregulated (left, blue) and upregulated (right, red) genes are shown, and total numbers are depicted in the top corners. (**E**) Overlap of significantly differentially expressed genes between APE1_null1_ and APE1_null2_ mESC clones from RNA-seq in panel (D). Venn diagram illustrating the number of genes downregulated (lower, blue) and upregulated (upper, red) meeting the significance threshold (log_2_FC > ±1, FDR < 0.05). (F) Metabolic activity assays (WST-8) were used as indicator of mESC survival after treatment with different concentrations of methyl methanesulfonate (MMS) (**F**), menadione (**G**), or camptothecin (CPT) (**H**) in wt, APE1_null_, and XRCC1_null_ mESCs. Relative absorbance measurements normalized to untreated controls are visualized. Shown are results of three independent experiments with technical duplicates with standard errors. (**I**) Genome-wide abundance of 5mC, 5hmC, 5fC, and 5caC in wt and APE1_null_ mESCs measured in duplicates by HPLC-MS/MS. (**J**) Global levels of 5fC and 5caC in wt and APE1_null_ mESCs with and without induction of TDG knockout measured by HPLC-MS/MS. Bars represent means of biological duplicates or triplicates. For this experiment, each cell line was measured once, and genotypically identical clones were grouped for analysis. Data for APE1_null2_ are not available. (**K**) Upper panel: Representative immunoblot of TDG, TDG-SUMO (detected with the same antibody) with Tubulin as loading control, of whole-cell extracts of mESC lines indicated. Lower panel: Quantitation of immunoblots (*n* ≥ 4) showing relative TDG expression normalized to Tubulin expression. Shown are means with standard errors. Asterisks indicate *P*-values of unpaired, two-tailed Student’s *t*-test. *P ≤ .05, **P ≤ .01, ***P ≤ .001, and ^****^P ≤ .0001.

To facilitate a systematic investigation of APE1 in DNA repair and active DNA demethylation, we generated constitutive APE1 knockout (APE1_null_) mESCs using CRISPR-Cas9 technology. Four clones were derived from two different wt mESC isolates, APE1_null1/2_ from wt_1_ and APE1_null3/4_ from wt_2_. Both originate from a parental mESC line equipped with an inducible TDG depletion cassette [37] and represent independent isolates generated during later kanamycin-resistant cassette removal. Sequencing of the DNA region surrounding the two gRNAs confirmed gene-disruptive sequence alterations at the *Ape1* locus in all APE1_null_ clones (Supplementary Fig. 1A). Immunoblotting, immunofluorescence, and reverse transcription quantitative polymerase chain reaction (RT-qPCR) of RNA confirmed the complete absence of APE1 protein and highly reduced transcript levels, respectively, in all knockout clones (Fig. 1A and Supplementary Fig. 1B and C). Phenotypically, the four isolates showed some differences across the assays described below. To provide a visual impression on their phenotypic profiles, we summarized all measured traits in a heatmap, scaled to relative differences from wt_1_ (Supplementary Fig. 1D). Analysis of the cell doubling time revealed variability among the clones, with APE1_null1_ exhibiting a significantly shorter doubling time compared to other APE1_null_ clones (Fig. 1B). Despite this difference, all APE1_null_ mESC lines exhibit no significant changes in cell cycle distribution (G1, S, and G2/M phases) (Fig. 1C). To assess the impact of *Ape1* deletion on global gene expression, we performed ribo-depleted total RNA sequencing (RNA-seq) in wt_1_, APE1_null1_, and APE1_null2_ mESCs cultured in 2i medium. Differential gene expression analysis revealed a substantial transcriptional response to APE1 loss, with hundreds of significantly deregulated transcripts (Fig. 1D and Supplementary Fig. 1E).

APE1 loss may affect transcription through several potential mechanisms, including the disruption of its redox regulatory function, impairment of active DNA demethylation, or the accumulation of AP sites in transcribed regions [45]. Given that the accumulation of AP sites could distort transcript quantification overall, we investigated whether APE1_null_ mESCs exhibit evidence of premature transcription termination in our RNA-seq data. To test this, we analyzed read coverage and normalized each transcript 5′–3′ across the longest transcript per gene (Supplementary Fig. 1F). Coverage profiles were nearly identical between wt_1_ and APE1_null_ mESCs until approximately the 70th percentile, with only a slight drop in read density at the transcript ends in APE1_null_ mESC clones. This finding suggests that the broad alterations in gene expression following APE1 loss are more likely attributable to a targeted cellular response than to damage-induced abortive transcription. Regarding differential expression, APE1_null1_ displays twice (1003) as many downregulated genes than APE1_null2_ (512) when compared to wt mESCs, while both APE1_null_ clones show similar numbers of upregulated genes (∼900; Fig. 1E). The overlap of differentially expressed genes (448 up or 283 down) between APE1_null1_ and APE1_null2_ is considerable, and Gene Ontology analyses revealed that both gene sets are associated with largely overlapping molecular pathways, enriched in categories related to transcriptional regulation (Supplementary Fig. 1G and H). Despite this overlap, each cell line also displayed a distinct set of differentially expressed genes, suggesting a variability in cellular responses to APE1 deficiency. We therefore conclude that APE1 deficiency causes a global transcriptional deregulation. The underlying mechanisms remain unclear but are likely multifactorial, potentially involving impaired APE1-mediated redox regulation of transcription factors, disruption of the DNA methylation cycle, and/or indirect cellular stress responses.

APE2 is the second known mammalian AP endonuclease [26, 46] with distinct and non-redundant functions from APE1. We nevertheless examined APE2 for potential compensatory upregulation in response to APE1 absence. While APE2 transcript levels were not significantly altered in the RNA-seq analysis performed, we found the APE2 protein to be reduced in two of the four APE1_null_ clones (Supplementary Fig 1I). As no physical interaction or regulatory relationship between APE1 and APE2 has been reported and the downregulation of APE2 occurs randomly in our clones, we conclude that APE2 does not systematically compensate for APE1 loss in mESCs.

To further characterize the APE1_null_ clones, we assessed their sensitivity to genotoxic agents that induce different types of DNA damage. MMS was applied to induce alkylating DNA base lesions, menadione to generate oxidative DNA base damage, while CPT, a topoisomerase I inhibitor (TOP1i), to provoke replication stress and replication-associated DSBs [47–49]. As a readout for cell viability, we used the WST-8 assay, measuring metabolic activity. We observed that the loss of APE1 sensitizes mESCs dramatically to MMS (Fig. 1F and Supplementary Fig. 1J). To compare the impact of a BER defect at the DNA strand incision step created by APE1 with a downstream defect in SSB repair, we also tested XRCC1_null_ mESCs [36], which show similar sensitivity to MMS as APE1_null_ mESCs (Fig. 1F). Consistently, treatment of wt mESCs with MMS in combination with the APE1 endonuclease inhibitor CRT0044876 (CRT) resulted in heightened sensitivity compared to MMS alone, indicating that APE1’s catalytic activity is essential for repairing alkylation-induced DNA damage (Supplementary Fig. 1K). Interestingly, at the lowest dose of menadione, the mESCs responded with an increase in metabolic activity, despite notable cellular stress by morphological observation (data not shown). This can be explained by a functional oxidative stress response that enhances cellular antioxidant pathways and/or the boosting of metabolic activity through recycling of reactive oxygen species [50]. At higher concentrations of menadione, we observed significant hypersensitivity for all APE1_null_ clones except APE1_null1_ (Fig. 1G and Supplementary Fig. 1L). XRCC1_null_ mESCs were drastically more sensitive to menadione treatment than the APE1_null_ mESCs (Fig. 1G). No significant differences were observed between APE1_null_ and wt mESCs treated with CPT, whereas XRCC1_null_ mESCs demonstrated a higher sensitivity to CPT at a lower concentration used (Fig. 1H and Supplementary Fig. 1M). In summary, all APE1_null_ mESCs show marked sensitivity to MMS-induced alkylating DNA damage, whereas only three of the four APE1_null_ mESCs also display increased sensitivity toward oxidative stress while all maintain normal resistance to TOP1i-induced DNA damage. We conclude that APE1 contributes to efficient repair of alkylating and oxidative DNA base damage but is not engaged in the repair of DNA DSBs accumulating upon TOPi.

To assess the impact of *Ape1* deletion on the genome-wide abundance of TET-generated 5mC derivatives 5hmC, 5fC, and 5caC, we measured their levels via HPLC-MS/MS. In APE1_null_ mESCs, 5mC and 5hmC levels were significantly reduced when compared to wt mESCs, while 5fC and 5caC levels showed a mild trend for an increase (Fig. 1I). Depletion of TDG caused an increase of 5fC and 5caC, as observed previously, irrespective of the APE1 status (Fig. 1J). These findings are consistent with TDG processing 5fC and 5caC intermediates of active DNA demethylation upstream of APE1 and independently of the presence of APE1. Notably, immunoblotting revealed a mild reduction in TDG protein levels in APE1-deficient mESCs (Fig. 1K and Supplementary Fig. 1N), potentially accounting for the modest elevation in 5fC and 5caC levels.

Taken together, these results demonstrate that APE1 is not essential for survival of mESCs in unchallenged conditions, but its deletion leads to increased sensitivity toward alkylating and oxidative DNA damage. No increased sensitivity to TOP1i-induced replication stress was observed, indicating that APE1 loss does not cause a general DNA repair defect. APE1’s loss does impact levels of 5mC and 5hmC, suggesting that the cycle of dynamic methylation is disturbed in APE1_null_ mESCs. APE1 deficiency, however, does reduce TDG expression, which may be an adaptive response to limit the AP site load in mESCs with high TET activity.

### APE1 processes AP sites generated by conventional BER and by active DNA demethylation and is essential for mESC differentiation in early mouse development

**Figure 2.**
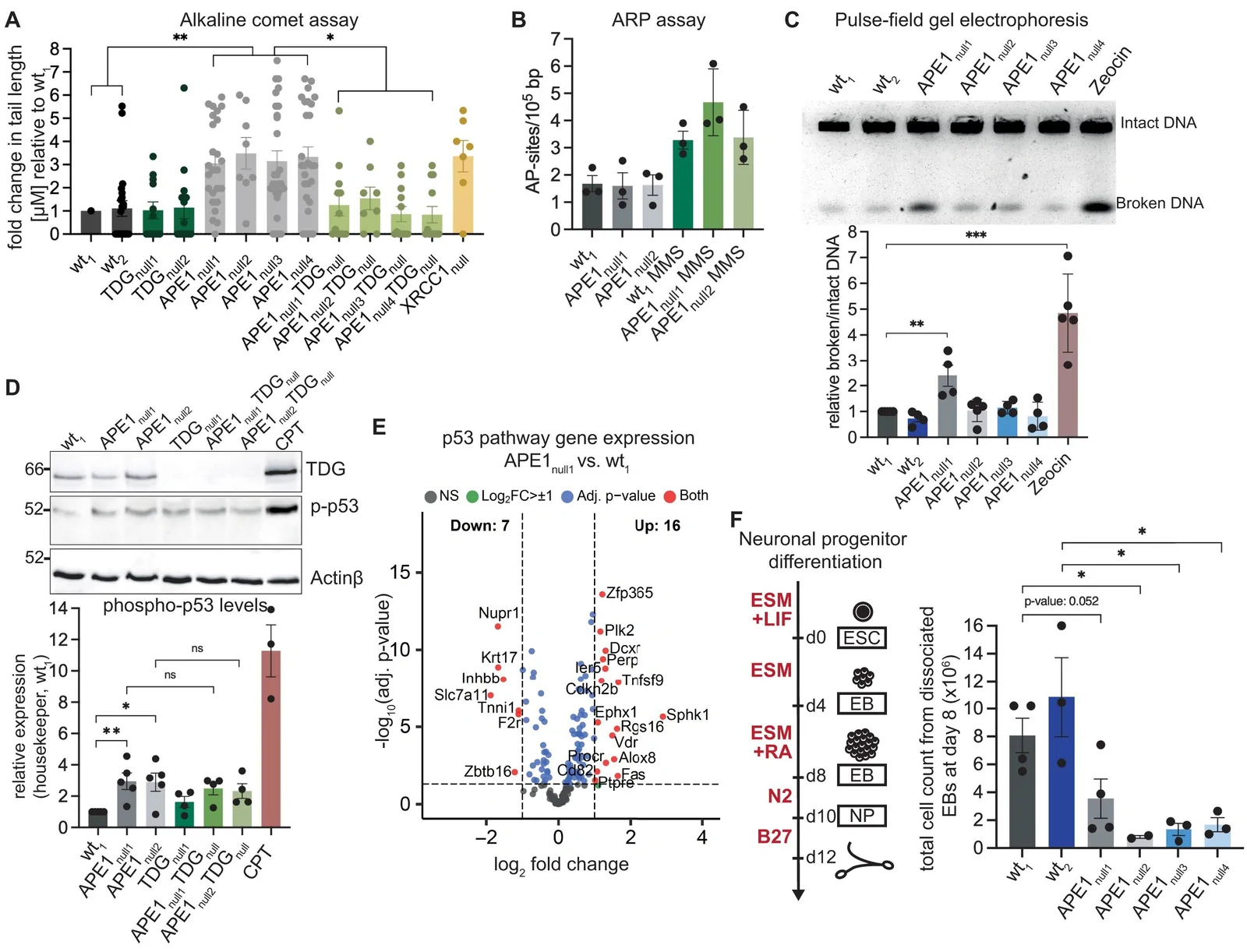
APE1 depletion increases DNA damage in a partially TDG-dependent manner and disrupts neuronal progenitor (NP) formation. (**A**) Tail lengths derived from alkaline comet assays were measured for wt, TDG_null_, APE1_null_, APE1_null_ TDG_null_, and XRCC1_null_ mESC lines and presented as mean fold changes (with standard errors) relative to wt_1_. Measured were *n* > 100 cells per *n* > 7 biological replicates. (**B**) Genome-wide AP site quantitation in wt and APE1_null_ mESCs using an ARP-assay. Data shown for untreated mESCs and cells treated with 1 mM MMS for 1 h. (**C**) Upper panel shows representative image of pulse-field gel electrophoresis (PFGE) of mESC clones indicated. 20 µg/ml Zeocin was used for 24 h as positive control. Lower panel shows quantitation of PFGEs, such as in upper panel, with means and standard error of at least four independent experiments. (**D**) Upper panel shows representative immunoblot of phospho-p53 (p-p53; serine 15), TDG, and Actinβ as loading control of whole-cell extracts of mESC lines indicated. 50 µM CPT was used for 24 h as positive control for p53 phosphorylation. Lower panel presents quantitation of p-p53 immunodetection, as in upper panel, of *n* ≥ 3 independent experiments with means and standard error shown. (**E**) Volcano plot of differentially expressed genes from the gene set enrichment analysis (GSEA) Hallmark p53 pathway in APE1_null1_ mESCs versus wt_1_ mESCs. Significantly (log_2_FC > ±1, FDR < 0.05) downregulated and upregulated genes are labelled, and total numbers are depicted in the top corners. (**F**) NP differentiation protocol and cell count of dissociated embryoid bodies (EB) after 8 days of differentiation of indicated mESC lines. Means and standard errors are shown for at least two independent experiments. ESC, embryonic stem cells; EB, embryoid body; NP, neuronal progenitor; ESM, embryonic stem cell medium; LIF, leukemia inhibitory factor; RA, all trans-retinoic acid; N2/B27, medium supplements. Asterisks indicate *P*-values by unpaired, two-tailed Student’s *t*-test. *P ≤ .05, **P ≤ .01, and ***P ≤ .001.

To investigate the contribution of APE1 to the repair of spontaneous DNA damage, we quantitatively assessed DNA damage in mESCs cultivated in 2i medium, i.e. in a state of naïve pluripotency. Using single-cell electrophoresis (comet assay) under alkaline conditions to monitor AP sites and DNA breaks simultaneously, we observed a significant increase in tail length in APE1_null_ mESCs, with damage levels elevated by ∼3-fold compared to wt mESCs (Fig. 2A). These findings demonstrate a critical role for APE1 in suppressing AP site and DNA break accumulation in mESCs. To investigate the contribution of TDG-dependent base excision on DNA damage accumulation in the absence of APE1, we performed the same analyses in the background of an induced TDG knockout (TDG_null_). Strikingly, the additional depletion of TDG significantly reduced the levels of spontaneous DNA breaks in APE1_null_ mESCs close to the level observed in wt mESCs (Fig. 2A). This result shows that TDG-generated AP sites are a major contributor to DNA damage normally processed by APE1 in mESCs.

To specifically quantify genome-wide AP site levels, we employed an ARP-based assay and examined wt and APE1_null_ mESCs, both with and without MMS treatment. Unexpectedly, no significant differences were detected between genotypes under either condition (Fig. 2B and Supplementary Fig. 2A), while the AP site-inducing effect of MMS was clearly notable. Given the surprising nature of this observation, we independently confirmed the ARP results using click-fluoro-quant, a guanine-specific AP site detection assay [43]. Consistent with the ARP findings, this method detected no differences between wt and APE1_null_ mESCs (Supplementary Fig. 2B) and also detected no SSB elevation in APE1_null_ mESCs (data not shown). The discrepancy with comet assay results likely reflects its superior sensitivity for detecting DNA damage at the single-cell level. The absence of elevated AP site levels in APE1_null_ mESCs suggests the activation of efficient compensatory repair pathways. Meanwhile, the increased DNA damage observed in the comet assay may reflect repair intermediates generated by these alternative mechanisms or secondary lesions stemming from the inherent instability of AP sites, which are not detectable by methods like the ARP assay and click-fluoro-quant.

We next used PFGE to assess DSB levels in APE1_null_ and APE1_null_TDG_null_ mESCs. While three APE1_null_ clones showed no DSB accumulation compared to wt mESCs, one clone, APE1_null1_, showed a significant increase (Fig. 2C). Notably, the same clone also distinguished itself by a shorter doubling time (Fig. 1B). Depletion of TDG in wt, APE1_null1_, and APE1_null2_ mESCs had no additional effect on DSB levels (Supplementary Fig. 2C).

To assess whether the observed DNA damage accumulation in APE1-deficient mESCs activates a DDR, we analyzed the phosphorylation status of p53 (p-p53) by immunoblotting. We conducted this experiment in two APE1_null_ clones (APE1_null1_ and APE1_null2_), taking APE1_null2_ as representative for APE1_null3_ and APE1_null4_, based on the overall similarity of these clones. Both APE1_null_ mESC lines show slightly but significantly elevated levels of p-p53 when compared to wt mESCs (Fig. 2D and Supplementary Fig. 2D). The additional depletion of TDG did not affect p53 phosphorylation in APE1_null_ mESCs. Yet, TDG_null_ mESCs showed a slightly higher basal level of p53 phosphorylation, indicating that p53 activation by APE1 loss is less pronounced in the absence of TDG. Consistent with p53 activation, GSEA on the RNA-seq data revealed a statistically significant upregulation of the p53 pathway (Hallmark dataset) in APE1_null1_ mESCs (*P*-value = .022; Supplementary Fig. 2F, left panel) but not in APE1_null2_ (Supplementary Fig. 2F, right panel). Nevertheless, several transcripts from the gene set were significantly deregulated in both APE1_null1_ (Fig. 2E) and APE1_null2_ mESCs (Supplementary Fig. 2E), with substantial overlap between the two clones. Specifically, we observe altered expression of genes involved in stress response and cell survival (*Slc7a11, Ephx1, Dcxr, Nupr1, Sphk1, Ier5, Zfp365*), cell cycle regulation (*Cdkn2b, Plk2*), and apoptosis (*Fas, Perp*), primarily in upregulated but also in downregulated transcripts in APE1_null1_ as well as in APE1_null2_ mESCs. While APE1 is known to modulate p53 activity through its redox function, the observed increase in p-p53 levels, along with the consistent transcriptional changes in p53 network genes, suggests that the observed stress response is primarily driven by DNA damage-induced activation of p53.

While mESCs commit to differentiation, an apparent paradox emerges: global DNA methylation levels rise, yet the activity of TET-TDG-mediated active DNA demethylation ramps-up in parallel. This duality reflects the dynamic remodeling of the epigenome and is measurable by an increase of 5fC and 5caC (steady-state levels) from ∼60 000 to ∼95 000 per genome upon induction of cell differentiation [35, 36]. To investigate the involvement of APE1 in mESC differentiation, we tested the ability of APE1_null_ mESCs to progress through neuronal progenitor (NP) differentiation via embryoid body (EB) formation [38]. While TDG and the BER scaffold protein XRCC1 were shown to be required for full neuronal lineage commitment in culture, both of them are dispensable for the initial EB formation step [36]. By contrast, APE1_null_ mESCs showed significant cell death already during EB formation as evident from reduction in cell counts on day 8 of differentiation when compared to wt mESCs (Fig. 2F and Supplementary Fig. 2G). This drastic loss of cells in APE1_null_ cultures prohibited the continuation of NP formation under standard culture conditions. Using small dish sizes, however, allowed some formation of NPs and neuronal networks, although with poor efficiency (Supplementary Fig. 2G).

Altogether, these findings demonstrate that endogenous DNA damage accumulates and activates a low-level, p53-dependent damage response in unchallenged APE1_null_ mESCs. A significant fraction of this damage is dependent on the presence of TDG, establishing the involvement of APE1 in active DNA demethylation. Like TDG- and XRCC1-deficient mESCs, APE1_null_ mESCs fail to efficiently differentiate toward the neuronal lineage. However, unlike TDG and XRCC1 depletion, APE1 protects from massive cell loss already at the stage of EB formation, which we interpret to be a consequence of an overload of AP sites generated by active DNA demethylation.

### Genome-wide fitness screen reveals critical pathways in APE1_null_ mESCs

**Figure 3.**
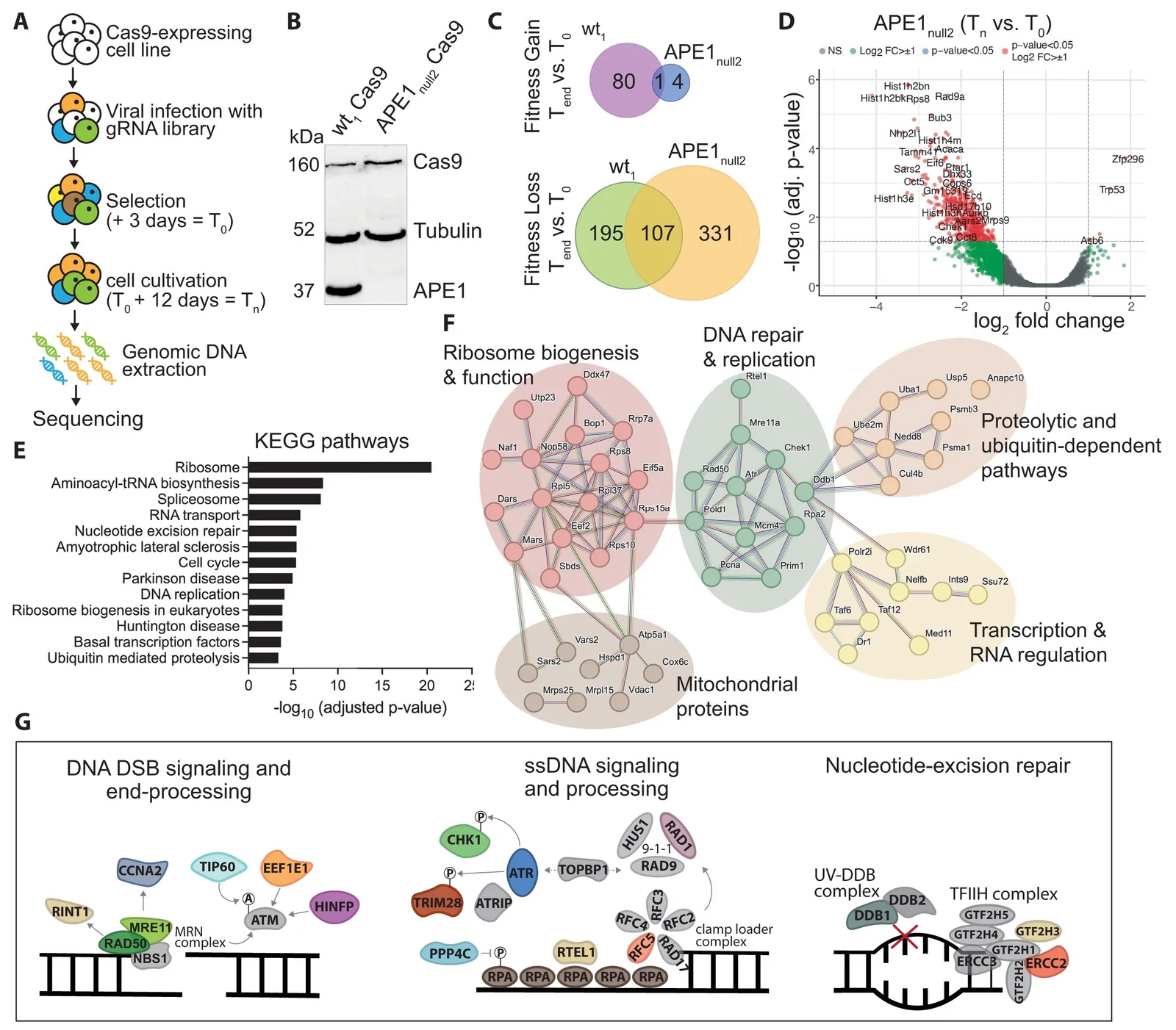
Genome-wide fitness screen highlights key pathways and vulnerabilities in APE1_null_ mESCs. (**A**) Schematic of the genome-wide CRISPR/Cas9 knockout screen in mESCs. (**B**) Immunoblot showing Cas9 and APE1 expression in whole-cell extracts of the newly generated mESC lines indicated, with Tubulin serving as loading control. (**C**) Venn diagram illustrating the overlap of significantly enriched (fitness gain) or depleted (fitness loss) CRISPR screen gene-hits between wt_1_ and APE1_null2_ mESCs (log_2_FC > ±1, FDR < 0.05). (**D**) Volcano plot showing CRISPR screen gene-hits with significant changes in fitness in APE1_null2_ mESCs between end timepoint *T*_n_ versus start timepoint *T*_0_ (log_2_FC > ±1, FDR < 0.05). (**E**) Kyoto Encyclopedia of Genes and Genomes (KEGG) pathway enrichment analysis of APE1-specific hits. (**F**) Classification of the APE1-specific hits into functional clusters using STRING. The figure shows representative genes for each cluster only. (**G**) Schematic representation of APE1-specific vulnerability hits associated with different DDR and repair pathways, identified in the CRISPR screen. Colored proteins represent significant APE1-specific hits, while gray proteins serve to complete the depicted pathways.

To further explore functional interactions with APE1, we conducted an unbiased genome-wide CRISPR/Cas9 knockout screen in wt and APE1_null_ mESCs (Fig. 3A). For this screening approach, we selected the APE1_null2_ mESC clone due to its phenotypic similarity to 3 out of 4 APE1_null_ clones (Supplementary Fig. 1D). To ensure uniform Cas9 expression, stable Cas9-GFP-expressing mESC lines were generated using lentiviral infection and subsequent selection for GFP expression. Cas9 expression was then validated by immunoblotting (Fig. 3B), and Cas9 activity was tested using a gRNA targeting GFP and flow cytometric quantitation of GFP-positive cells (Supplementary Fig. 3A). Cas9-positive mESC clones (APE1_null2_Cas9 and wt_1_Cas9) were transduced in triplicates with a genome-wide lentiviral CRISPR gRNA library [39]. mESCs were harvested 3 days after library transduction and selection to capture the initial representation of gRNAs (*T*_0_) and 12 days later (*T*_n_) to allow gRNA-induced proliferation effects to manifest. Library preparation and sequencing were performed on all samples from both time points, and significantly enriched (fitness gain) or depleted gRNAs (fitness loss) were identified by comparing gRNA representation between the two timepoints in both genotypes (log_2_FC > ±1, FDR < 0.05). The analysis of *T*_0_ samples shows no significant differences in gRNA distribution between wt_1_Cas9 and APE1_null2_Cas9 (Supplementary Fig. 3B), confirming equal initial gRNA representation in both clones. Examining the specific gene deletion effects over time within the same genotype, we identified 383 gene hits with significant fitness effects in wt mESCs (302 depleted, 81 enriched; Fig. 3C and Supplementary Fig. 3C) and 443 in APE1_null_ mESCs (438 depleted, 5 enriched; Fig. 3C and D). 108 gene hits overlapped between the two genotypes, while 335 gene hits were unique to APE1_null_ mESCs (Fig. 3C). Of the latter, four genes conferred a fitness gain, while 331 genes conferred a fitness loss specifically in absence of APE1 (APE1-specific hits).

Gene Ontology analysis with APE1-specific hits highlighted a variety of different molecular processes relating to DNA repair but also to transcription, translation, cell cycle, and to pathways associated with neurological diseases (Fig. 3E). Exploring the functional space of identified genes further, we categorized the APE1-specific hits into ten distinct functional clusters based on their known biological roles (Supplementary Fig. 3D). The five most dominant clusters by numbers of gene-hits detected were ribosome biogenesis and function, DNA repair and replication, transcription and RNA regulation, proteolytic and ubiquitin-dependent pathways, and mitochondrial proteins (Fig. 3F). The identification of vulnerability genes involved in ribosome biogenesis and function aligns with recent findings of a nucleolar localization of APE1 as well as its suggested role in ribosomal RNA quality control [51–53]. Similarly, the transcription and RNA regulation cluster together with the appearance of mitochondrial proteins are in line with APE1’s established functions in redox regulation of several key transcription factors, its endoribonuclease activity, and its translocation to mitochondria [54–56]. By contrast, the identification of genes related to proteolytic and ubiquitin-proteasome system introduces a new functional context, as no direct connection between APE1 and these pathways has been reported so far.

The DNA repair and replication cluster was an expected finding and offered a starting point for examining the mechanisms compensating for the loss of APE1 in mESCs. APE1-specific hits were widespread across DDR pathways and can be grouped into three functional categories: DNA DSB signaling and processing, single-stranded DNA (ssDNA) signaling and processing, and NER (Fig. 3G). The first category, DNA DSB signaling and processing, was defined by the identification of two members of the MRN complex (MRE11, RAD50), which detects DSBs, recruits and activates the central DDR kinase ATM [57], and contributes to DSB end-processing [58]. Additional factors associated with ATM or the MRN complex were also discovered, including TIP60, EEF1E1, HINFP, CCNA2, and RINT1 [59–64]. In the second category, we identified key factors involved in ssDNA signaling and processing, including the ssDNA-binding protein RPA2 and the effector kinase ATR, which is activated in response to ssDNA [65]. ATR activation involves the 9-1-1 complex, which is loaded onto DNA by the clamp loader complex that recognizes ssDNA–dsDNA junctions [66], a process involving RAD1 and RFC5, which we identified as APE1-specific hits. Moreover, two prominent phosphorylation targets of ATR, CHK1 and TRIM28, were detected [67], as well as two proteins that associate with RPA. There were the protein phosphatase (PPP4C), which dephosphorylates RPA to facilitate downstream repair [68], and the DNA helicase RTEL1, which interacts physically with RPA and acts as an inhibitor of HR by resolving strand invasion structures (e.g. D-loops) [69–71]. Notably, other genes involved in ssDNA signaling and processing (Fig. 3G, shown in gray) also caused a fitness defect upon deletion in APE1_null_ mESCs, though their effect was not statistically significant. The third category, NER, includes three factors identified as APE1-specific hit: DDB1, a component of the UV-DDB complex (DDB1–DDB2), plays a key role in recognizing DNA damage and initiating repair [72, 73], while GTF2H3 [74] and the helicase XPD [75], both subunits of the TFIIH complex, facilitate DNA unwinding and lesion verification. These proteins are core components of NER and thus central to the repair of bulky DNA lesions and AP sites [76].

Taken together, the CRISPR screen identified a wide range of molecular pathways that compensate for an APE1 loss in mESCs. Among them are several DDR factors, implicating the need for engagement of a network of different activities to overcome the potentially harmful BER intermediates accumulating in APE1_null_ mESCs.

### APE1-deficient mESCs depend on ATM to signal accumulated DNA damage

**Figure 4.**
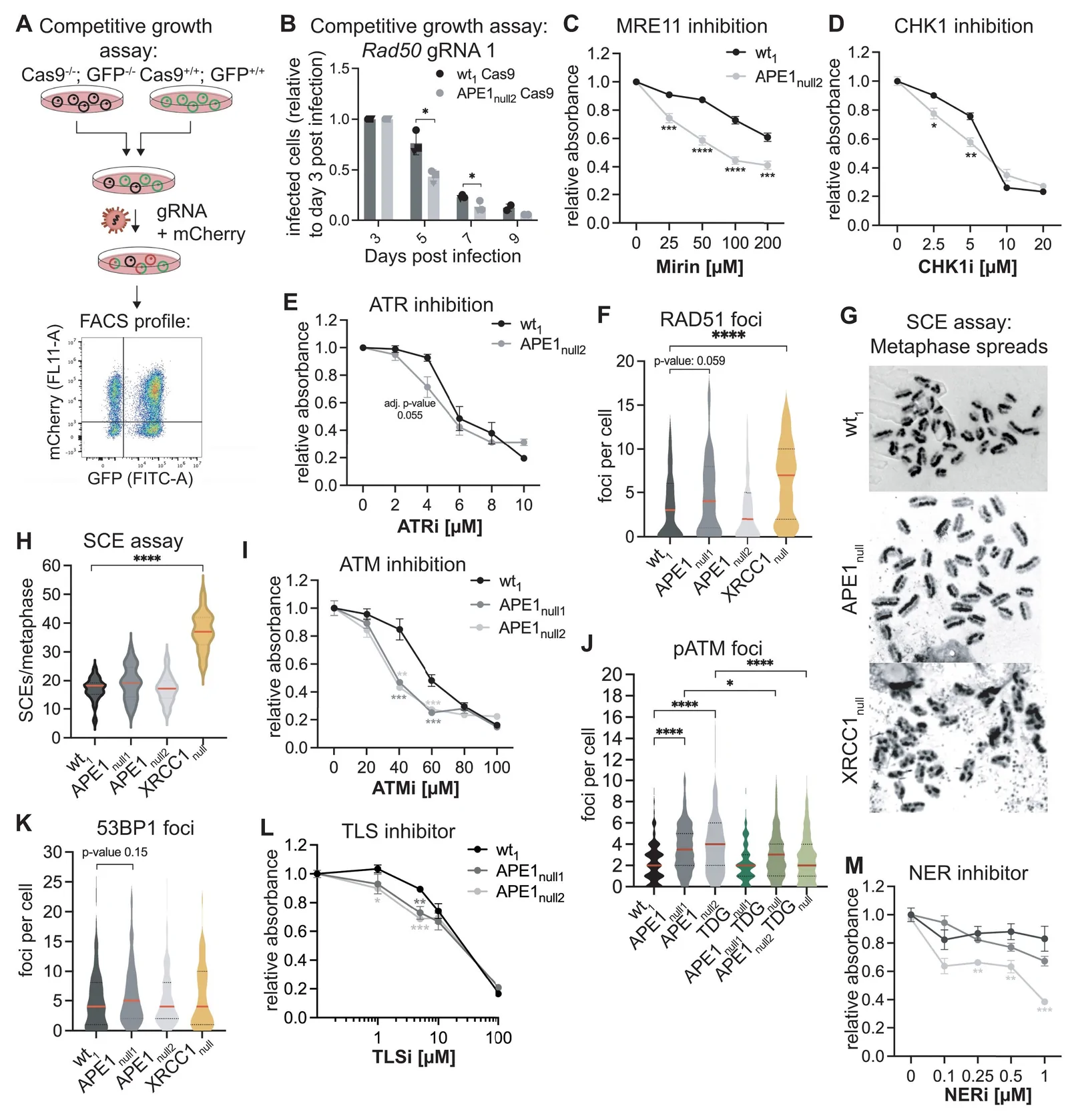
APE1_null_ mESCs depend on a complex network of DDR and repair activities. (**A**) Schematic of the flow cytometry-based competitive growth assay based on lentiviral infection, used to validate APE1-specific hits from the CRISPR screen. (**B**) Competitive growth assay using gRNA 1 against *Rad50* to validate CRISPR screen results. Shown are the cell counts of wt or APE1_null_ mESCs over time, normalized to scrambled gRNA infection and to the starting timepoint (day three post infection). Displayed are measurements of individual experiments and means of three independent experiments with standard errors. (C) Metabolic activity assay (WST-8) performed on wt_1_ and APE1_null2_ mESCs exposed to different concentrations of small-molecule inhibitors targeting MRE11 (mirin) (**C**), CHK1 (rabusertib, CHK1i) (**D**), and ATR (VE-822, ATRi) (**E**). Shown are mean values normalized to untreated controls; standard errors are from three independent experiments with technical duplicates each. (**F**) Quantitation of RAD51 foci using immunofluorescence in mESC lines indicated. *n* = 3 slides with >110 individual mESCs analyzed. Kruskal–Wallis test with following post-hoc pairwise comparison was performed using Dunn’s multiple comparison test. (**G**) Representative images of sister chromatid-exchange (SCE) assay, showing metaphase spreads and differentially stained sister chromatids of indicated mESC lines. (**H**) Quantitation of SCEs in mESC lines indicated. *n* ≥ 33 metaphases were analyzed. (**I**) Effects of ATM inhibitor (KU55933, ATMi) at different concentrations in a metabolic activity assay (WST-8) comparing wt and APE1_null_ mESCs. Shown are the relative absorbance measurements, normalized to untreated controls; standard errors are from three independent experiments with technical triplicates. (**J**) Quantitation of p-ATM foci detected in mESC lines indicated, measured via immunofluorescence. *n* = 3 slides with >150 cells analyzed. Kruskal–Wallis test with following post-hoc pairwise comparison was performed using Dunn’s multiple comparison test. (**K**) Quantitation of 53BP1 foci detected in mESC line indicated, measured via immunofluorescence. *n* = 3 slides with >150 cells analyzed. Kruskal–Wallis test with following post-hoc pairwise comparison was performed using Dunn’s multiple comparison test. (**L**) TLS inhibitor (JH-RE-06, TLSi) was used at concentrations of 0.1, 1, 5, 10, and 100 µM for 24 h of wt and APE1_null_ mESCs. Shown are the relative absorbance measurements from a metabolic assay (WST-8), normalized to untreated controls. Standard errors are from three independent experiments with technical duplicates. (**M**) NERi (NSC130813) was used at concentrations indicated for 24 h for wt and APE1_null_ mESCs. Shown are the relative absorbance measurements from a metabolic assay (WST-8), normalized to untreated controls. Standard errors are from two independent experiments with technical quadruplets. Asterisks indicate *P*-values of unpaired, two-tailed Student’s *t*-test, or indicated statistical tests as indicated. *P ≤ .05; **P ≤ .01, ***P ≤ .001, and ^****^P ≤ .0001.

Following up on the CRISPR screen results, we focused on deciphering the DDR processes that compensate for the loss of APE1 in mESCs. The spectrum of DDR genes providing fitness to APE1_null2_ mESCs included *Mre11a, Rad50, Rpa2, Atr, Chek1*, and *Ddb1*. These genes encode central components of DNA damage signaling, DNA double-strand break repair (DSBR), and NER. To validate these hits, we developed a flow cytometry-based competitive growth assay, generating lentiviruses carrying a gRNA targeting the gene of interest as well as an mCherry reporter gene (Fig. 4A). In this assay, the depletion of *Rad50*, an essential gene [77], reduced the fitness of wt_1_ mESCs as expected, while APE1_null2_ mESCs exhibited markedly faster cell loss, thus validating the synthetic sensitivity of co-depletion of APE1 and RAD50 (Fig. 4B and Supplementary Fig. 4A). In the same way, we validated the synthetic sickness of *Ape1* with *Rpa2* and *Ddb1*. Like *Rad50*, these genes are essential [78–80] and strongly reduce fitness in wt mESCs when depleted. We did observe a consistent trend for enhanced cell death when *Ddb1* or *Rpa2* were targeted in APE1-deficient mESCs (with two separated gRNAs each), but the difference to wt mESCs did not reach statistical significance (Supplementary Fig. 4B and C).

We used small-molecule inhibitors against MRE11 (mirin), CHK1 (rabusertib, CHK1i), and ATR (VE-822, ATRi) to validate these APE1-specific hits in a mESC survival assay. These experiments demonstrated a dose-dependent hypersensitivity of APE1_null2_ to the inhibition of MRE11 and CHK1 and, to a lesser extent, ATR (Fig. 4C–E). To assess the consistency of these findings across APE1_null_ mESC clones, we repeated small-molecule inhibitor treatments (mirin, CHK1i and ATRi) in the three additional APE1_null_ clones (Supplementary Fig. 4D–F). The results for APE1_null3_ and APE1_null4_ largely aligned with the findings for APE1_null2_ (Fig. 4C–E), demonstrating elevated sensitivity to the inhibition of MRE11 and CHK1, while the response to ATR inhibition was more variable. Notably, APE1_null1_ continued to show a distinct behavior, not showing any sensitivity toward mirin or ATRi and only very limited to CHK1i. To assess whether the catalytic activity of APE1 underlies the observed phenotypes, we co-treated wt mESCs with the APE1 endonuclease inhibitor CRT0044876 (CRT), either alone or in combination with mirin, CHK1i, or ATRi. The combination of CRT and mirin caused uncontrolled precipitation in the mESC culture medium, leading to unreliable results. Consequently, this condition was excluded from further analyses. The combination of CRT and CHK1i enhanced the sensitivity of wt mESCs, demonstrating that APE1’s endonuclease activity contributes to the synthetic lethality between APE1 and CHK1 (Supplementary Fig. 4G). By contrast, co-treatment with ATRi and CRT did not produce an additive effect (Supplementary Fig. 4H). This suggests that either the functional interaction between APE1 and ATR is independent of APE1’s catalytic activity, or the potent effect of ATRi masks any additional sensitivity conferred by CRT.

Among the APE1-specific hits tested, the MRE11 and RAD50 depletion showed the strongest APE1-dependent fitness reduction in mESCs (Fig. 4B and C; Supplementary Fig. 4A). Both proteins are key components of the MRN complex and are well-known for their role in DSB recognition, signaling, and processing [57]. We therefore investigated DSBR activity, specifically HR activity, in APE1-deficient mESCs further. HR dependency has previously been observed in BER-deficient cells, including XRCC1-deficient [31, 81] and PARP1-deficient cells [30, 82]. We assessed HR activity by quantitation of RAD51 foci and the frequency of sister chromatid exchanges (SCEs) in wt, APE1_null_, and XRCC1_null_ mESCs, whereby the latter serves as a control. Against our expectations, RAD51 foci counts were not elevated in APE1_null_ mESCs when compared to wt mESCs, whereas XRCC1_null_ mESCs showed the expected increase (Fig. 4F and Supplementary Fig. 4N). Similarly, APE1_null_ mESCs showed no significant increase in SCE events when compared to wt mESCs (∼18 SCEs/metaphase), whereas XRCC1_null_ mESCs did show the expected significant increase (∼40 SCEs/metaphase; Fig. 4G and H). These findings demonstrate that APE1_null_ mESCs depend on MRN complex to signal and possibly process DNA ends but do not engage in HR-mediated recombinational repair. These results clearly separate the phenotype of APE1-deficient mESCs from that of XRCC1-deficient mESCs, which strongly depend on the engagement of the full HR-pathway for DSB repair.

In addition to its critical role in DSB detection and processing, the MRN complex is also known for recruiting ATM to DSBs and promoting its activation by autophosphorylation [83]. We therefore tested the sensitivity of APE1_null_ mESCs to ATM inhibition using the KU-55933 inhibitor (ATMi) and observed a significant and dose-dependent decrease in mESC survival over 24 h in all APE1_null_ mESCs when compared to wt mESCs (Fig. 4I and Supplementary Fig. 4I). Consistently, immunofluorescence analysis showed elevated numbers of p-ATM foci in APE1_null_ mESCs (Fig. 4J and Supplementary Fig. 4P). ATM inhibition thus creates a critical vulnerability in absence of APE1, even though ATM itself did not emerge as an APE1-specific significant dropout hit in the CRISPR knockout screen. To determine whether the increased ATM activation in APE1_null_ mESCs was influenced by TDG activity, we examined pATM foci upon additional depletion of TDG. TDG loss led to a clear reduction in pATM foci, suggesting that TDG-mediated active DNA demethylation generates BER intermediates that trigger ATM activation, unless repaired by APE1 (Fig. 4J and Supplementary Fig. 4P). We then examined the effect of inhibition of MRE11 (Supplementary Fig. 4J), ATM (Supplementary Fig. 4K), CHK1 (Supplementary Fig. 4L), and ATR (Supplementary Fig. 4M) on the survival of APE1_null_ mESCs with or without TDG depletion. Contrary to the effect on pATM foci, TDG depletion did not significantly alter the sensitivity of APE1_null_ mESCs to these inhibitors after 24 h of treatment.

While the CRISPR screen identified early-acting factors in HR, the absence of increased RAD51 foci or SCEs in APE1_null_ mESCs suggests that DNA breaks accumulating are repaired through alternative pathways. We therefore expanded our analysis to further DDR mechanisms that can operate downstream of DNA end recognition and processing. Implicated as candidate pathways were NHEJ, TLS, and aspects of NER. First, we quantified 53BP1 foci, a marker indicating the engagement of NHEJ for DSB repair. 53BP1 foci formation was not different between wt and APE1_null_ mESCs, ruling out NHEJ as a major compensatory pathway for APE1 deficiency (Fig. 4K and Supplementary Fig. 4O). To address a possible involvement of TLS, a DNA damage tolerance mechanism acting at DNA polymerase-blocking lesions such as AP sites, we used JH-RE-06, a potent TLSi that disrupts the interaction between REV1 and REV7, which is essential for polymerase-dependent lesion extension. In a metabolic activity assay, we observed a moderate but statistically significant hypersensitivity of APE1_null_ mESCs to JH-RE-06 when compared to wt mESCs (Fig. 4L). Similarly, we tested the effect of NSC130813, an NERi that disrupts the interaction between two key NER factors XPF and ERCC1. In the metabolic activity assay, APE1_null2_ mESCs show a significant hypersensitivity to NERi, while again APE1_null1_ mESCs did not (Fig. 4M). These findings suggest that both NER and TLS can contribute to the survival of mESCs in the absence of APE1.

Taken together, our results demonstrate vital roles for the MRN complex and ATM signaling, but not for HR or NHEJ, in APE1-deficient mESCs. The observed reduction of ATM activation upon TDG depletion supports the notion that TDG-generated AP sites by 5fC and 5caC excision are a significant source of AP sites and SSBs and, hence, DNA damage signaling in APE1_null_ mESCs. Notably, the distinct behavior of APE1_null1_ clone shows that mESCs can take different routes to adapt to the loss of the major AP-endonuclease.

## Discussion

To investigate the role of the major AP-endonuclease APE1 in a cell system where both general base damage and TET–TDG-mediated DNA demethylation contribute to AP site formation, we examined the phenotypes and vulnerabilities associated with an APE1 defect in mESCs. We explored the specific contribution of AP sites arising from TDG-mediated active DNA demethylation and provide the first genetic evidence for APE1 processing AP sites in this pathway. Performing a genome-wide CRISPR screen for synthetic genetic interactions with APE1, we aimed to gain deeper insight into the complex functional network of AP site repair, including the compensatory mechanisms that support mESC viability in the absence of APE1, the major endonuclease that processes these lesions in mESCs.

A central question of this study was the extent to which TET–TDG-mediated active DNA demethylation contributes to the AP site load in mESCs and hence the phenotype observed in APE1-deficient mESCs. Our data document for the first time directly that TDG-mediated base excision accounts for a significant portion of AP sites and SSBs in APE1_null_ mESCs (Fig. 2A). This is evident from the significant reduction in DNA break levels (alkaline comet assay) and in pATM foci formation in APE1-deficient mESCs upon TDG depletion (Fig. 4J and Supplementary Fig. 4P). We therefore conclude that TDG-generated AP sites are repaired by APE1 and that efficient active DNA demethylation requires APE1 activity. This observation is further supported by the impaired differentiation of APE1_null_ mESCs into NP cells, a phenotype similarly observed in TDG- and XRCC1-deficient cells [36]. Specific to APE1-deficient mESCs, however, is the massive cell death occurring early in the differentiation process, i.e. during EB formation (Fig. 2F and Supplementary Fig. 2G). We propose that this phenotype arises from an increased burden of AP sites during TET–TDG-mediated active DNA demethylation, a process upregulated following differentiation priming. In this transition, steady-state levels of 5fC and 5caC rise from ~60 000 sites per pluripotent mESCs to around 95 000 in EBs [36]. In TET- and/or TDG-deficient cells, oxidized 5mC derivatives are not generated or excised, respectively, resulting in a failure of DNA methylation programming that will manifest itself only later in cell differentiation. Hence, whereas TET and TDG-deficient mESCs fail to differentiate due to their inability to dynamically change DNA methylation, APE1-deficient mESCs likely fail due to the accumulation of cytotoxic AP sites by active DNA demethylation. There is currently no other mechanism known that would contribute to elevated DNA base lesions during early differentiation and thereby explain the increased vulnerability of APE1_null_ mESCs to differentiation. In the absence of APE1, AP site repair is compromised, activating ATM-dependent DNA damage signaling and partially compensatory AP site repair mechanisms (Fig. 2A and 4J). Notably, TDG depletion did not improve the survival of APE1_null_ mESCs when alternative repair factors like MRE11 were inhibited. This observation suggests that AP sites arising from general base damage contribute to the reliance of mESCs on alternative AP site repair pathways. In summary, in APE1-deficient mESCs, TDG-dependent base excision significantly contributes to AP site burden and DNA damage signaling. However, depleting TDG fails to rescue cell viability when alternative repair pathways are impaired, underscoring the role of general base damage in this context.

The second core objective of this study was to map the functional interactome of APE1 in mESCs. The tolerance of mESCs to the loss of APE1 was an intriguing finding in itself, given that these cells continuously generate a relatively large number of AP sites by active DNA demethylation alone. Also, several previous attempts to generate APE1-deficient cells have failed, indicating a vital function of APE1 [19, 20], whereas it was successful in other cases [18, 22, 23, 26, 21, 24]. Therefore, the ability of cells to survive APE1 deficiency is determined by cell-type-specific features, particularly the presence and activity of compensatory pathways that can assume AP site processing functions in BER and/or SSB repair. Obvious backup mechanisms have been proposed to involve AP-lyase activities, such as provided by bifunctional DNA glycosylases or PARP1. These AP-lyases can incise AP sites and operate in combination with SSB end-processing enzymes like PNKP, TDP1, or APTXN to generate suitable 3′-OH SSB ends for repair, as usually performed by APE1 [25–28]. However, none of these obvious backup candidates for APE1 were identified as synthetic lethal partners of *Ape1* in our genome-wide CRISPR knockout screen. This may be explained by the fact that these enzymes alone fail to fully recapitulate the unique multifunctionality of APE1, which includes efficient AP site incision and 3′-end processing. Instead, we identified MRE11 as a nuclease with DNA end-processing activity, which is best known for its role in DSBR via HR. Notably, the concept of BER engaging MRE11 to cleave AP sites has been proposed for somatic hypermutation [84] and immunoglobulin gene conversion [85], involving an unconventional, mutagenic BER pathway. The fact that one APE1_null_ clone showed no sensitivity to MRE11 inhibition and also behaved differently in other assays illustrates that mESCs can activate alternative compensatory pathways in response to APE1 loss. We acknowledge here that stable CRISPR/Cas9-derived knockout cell lines are intrinsically limited by potential clone-specific adaptations, a limitation mitigable by RNA interference-based approaches. However, we deliberately chose this knockout strategy to comprehensively study the consequences of complete loss of APE1 and addressed clonal variability by analyzing multiple independent clones across most experiments. In the following, we will focus our discussion on the mechanistic implications of the phenotypes observed in the majority of clones.

**Figure 5.**
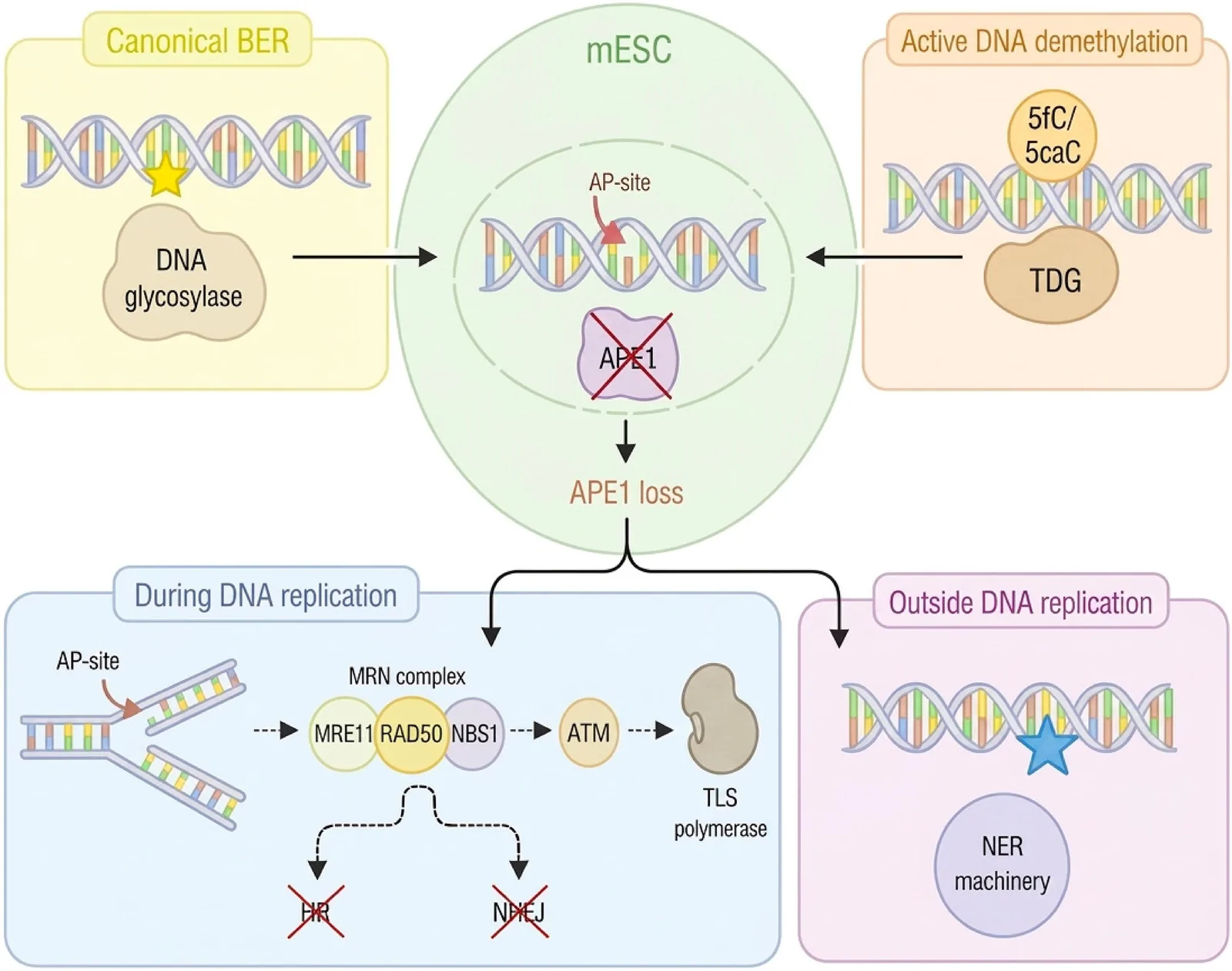
AP site repair in APE1_null_ mESCs. In the absence of APE1, AP site repair may follow distinct pathways depending on the genomic context. During DNA replication, unrepaired AP sites encountered by the replication fork can trigger the MRN–ATM signaling axis, which helps prevent DSB formation and facilitates replication progression through TLS. The two major DSBR pathways, HR and NHEJ are crossed out to indicate that while in replication stress, the MRN complex and ATM activities are often associated with HR activity, our data do not support the engagement of HR or NHEJ in this context. In non-replicating regions, including transcriptionally active chromatin or unengaged DNA, AP sites instead can be processed through NER.

Given that unresolved AP sites can convert into various other types of DNA lesions, including SSBs or DSBs, their persistence in the absence of APE1 may trigger a broad range of potential DNA repair pathways. HR has been shown to efficiently compensate for certain BER defects, affecting repair steps downstream of DNA strand incision. This is most prominently evidenced by the increase of SCEs in XRCC1-deficient cells [31, 81] or upon PARP1 inhibition or genetic loss [30, 82, 86, 87]. We identified HR-associated factors MRE11 and RAD50 as vulnerability hits in our CRISPR screen with APE1-deficient mESCs. Together, they form part of the MRN complex, which plays a central role in DNA end recognition and resection [58] and facilitates the recruitment and activation of the DNA damage signaling kinase ATM [83], also showing synthetic vulnerability upon APE1 loss (Fig. 4I and Supplementary Fig. 4I). We therefore reasoned that our APE1-deficient mESCs would engage the HR pathway and, consequently show elevated levels of SCEs or RAD51 foci. This, however, was clearly not the case (Fig. 2C and 4F–H; Supplementary Fig. 4N). Given this, it is important to note that all HR-associated proteins identified in the screen act upstream of the DNA strand exchange step, indicating that APE1_null_ mESCs primarily rely on the early steps of HR, including DNA damage recognition, signaling, and possibly resection, rather than on the complete pathway generating recombinant products. Further investigations are needed to clarify the precise role of these HR factors in compensating for APE1 loss, particularly whether these proteins function to facilitate the engagement of alternative repair pathways, independent of HR. NHEJ may be considered as such an alternative. However, we found no evidence for an increased engagement of NHEJ in APE1_null_ mESCs; no NHEJ factors were identified in the CRISPR screen and 53BP1 foci counts remained unchanged upon APE1 loss in mESCs (Fig. 4K and 5, Supplementary Fig. 4O). It is generally accepted, though, that AP sites stall the replication and transcription machineries [45, 88, 89], and that the MRN complex is recruited to stalled replication forks to aid their resolution (Fig. 5) [90–92]. Given the absence of increased recombination in APE1-deficient mESCs, the absence of a notable compensatory role of NHEJ in APE1-deficient mESCs, the vulnerability of these cells to the genetic depletion of NER proteins, and their hypersensitivity to inhibition of TLS or NER (Fig. 4L and M), we propose that unrepaired AP sites are processed via NER in non-replicating or transcribed DNA, and through MRN and TLS during DNA replication (Fig. 5). NER acts both, genome-wide through global genome repair (GG-NER) and also in transcribed regions via transcription-coupled NER (TC-NER) [93]. There is solid evidence for the UV-DDB complex, composed of DDB1 and DDB2, recognizing and binding AP sites [76, 94], and for core NER proteins, including XPF, XPG, and XPA, to either directly act on AP sites or otherwise promote AP site repair [29, 95]. Finally, there is ample evidence for a close relationship with a direct mechanistic interplay between BER and NER pathways [96–98]. Zharkov and colleagues investigated AP site repair in APE1-deficient HEK293FT cells and found that artificial AP site analogs could still be processed in the absence of APE1, strongly suggesting the existence of a backup repair mechanism [26, 21]. Consistent with our findings, they also reported no increase in AP site levels upon MMS treatment in the APE1-deficient HEK293FT cells, despite clear sensitivity to the agent. They identified a partial contribution of bifunctional DNA glycosylase NTHL1 to AP site repair, which was, however, not directly investigated in the APE1-deficient cells. While NER was not specifically tested, its involvement was considered unlikely, as it would have been detected with the experimental setup used [26]. NTHL1 did not emerge as vulnerability hit in our CRISPR screen, but we did identify several NER-associated proteins, including DDB1, GTF2H3, and XPG (Fig. 3G). Validation experiments with specific DDB1 depletion failed to reproduce a clear difference in survival between wt and APE1_null_ mESCs, which may be explained by the already pronounced sickness observed in wt mESCs (Supplementary Fig. 4B). However, we did measure an increased sensitivity of APE1-deficient mESCs to the treatment with a NERi (Fig. 4M). Further investigations are thus merited to mechanistically decipher the contribution of NER to AP site repair in the absence of APE1.

TLS provides a strategy to tolerate replication-blocking lesions such as AP sites (Fig. 5). In TLS, the replication machinery can switch to specialized TLS polymerases that insert nucleotides opposite the damaged base and extend from the distorted template. While this mechanism is often error-prone, it enables replication to continue without generating strand breaks or fork collapse [99]. Our observation of an increased sensitivity of APE1-deficient mESCs to a specific TLSi indicates that in the absence of APE1, unprocessed AP sites can be bypassed by TLS polymerases to prevent DNA replication collapse (Fig. 4L). This may be a way to limit DSB formation at the replication fork and, hence, to explain the absence of HR generated SCEs in APE1-deficient mESCs. Further experiments will have to clarify the mechanism underlying TLS mediated AP site repair/tolerance in APE1-deficient mESCs.

Collectively, our findings reveal an extended and coordinated interplay between NER, TLS, and DSBR pathways in AP site repair, illustrating how cells integrate multiple repair mechanisms to compensate for APE1 deficiency and sustain mESC viability.

## Supplementary Material

gkag921_Supplemental_Files

## Data Availability

The RNA-seq and CRISPR screen data generated in this study are available through the NCBI GEO repository (https://www.ncbi.nlm.nih.gov/geo) under accession numbers GSE295385 and GSE295386, respectively. The original data are stored on Zenodo at https://doi.org/10.5281/zenodo.15234066.

## Notes

Present address: Swiss RNA Mass Spectrometry Platform, Department of Chemistry, Biochemistry and Pharmaceutical Sciences, University of Bern, Bern 3012, Switzerland
