# Supplementary material for: Abasic site repair in APE1-deficient mouse embryonic stem cells: a matter for the MRN complex and ATM signaling
Source: Nucleic Acids Res. 2026 Sep 24;54(18):gkag921. doi: 10.1093/nar/gkag921 (PMC13601066; doi:10.1093/nar/gkag921)
Supplement: Supplementary file 1 [file gkag921_supplemental_files.zip › Hrovat et al 2026_Supplementary_Figures_260813.pdf]

SUPPLEMENTARY FIGURES

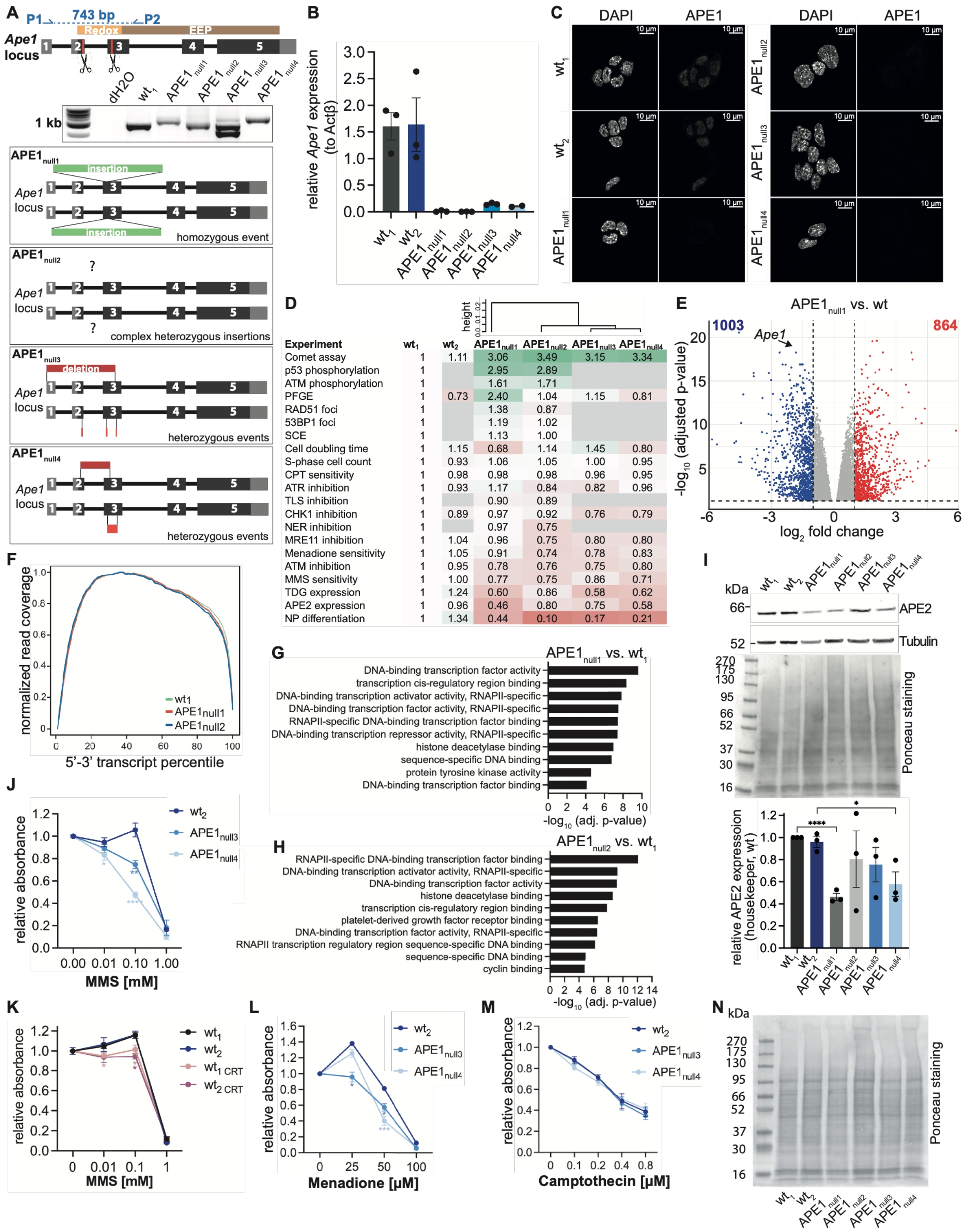

Supplementary Figure 1. APE1 knockout cell line characterization

*(**A**) Schematic of the Ape1 locus showing the targeting gRNAs (red and scissors) as well as the redox and the endonuclease-exonuclease-phosphatase (EEP) domain of Ape1. Blue arrows and dotted line indicate the positions of the diagnostic PCR primers used and the expected PCR product size. Results of the PCR amplification of the Ape1 locus from the genomic DNA of the mESC lines indicated are visualized in the panel below. The four lower boxes display schematically the identified sequence rearrangements of the four $APE1_{null}$ mESC lines. Deletions are highlighted by red markings, while insertions are highlighted by green markings. (**B**) RT-qPCR analysis of Ape1 expression at the exon 2/3 junction of the cell lines indicated. Three independent analyses were performed and shown with standard errors. (**C**) Representative immunofluorescence images of all mESC lines stained for APE1 (right panel) and counterstained with DAPI (left panel). (**D**) Summary heatmap of all experiments across all wt and $APE1_{null}$ mESC lines. Data from each experiment were normalized to wt_1 and visualized as a heatmap to illustrate relative effect sizes, with an accompanying dendrogram showing similarities among $APE1_{null}$ mESC clones. (**E**) Differential gene expression between $APE1_{null2}$ and wt mESC analyzed by total RNA-sequencing. Number of significantly ($log2FC > \pm 1$, $FDR < 0.05$) downregulated (blue) or upregulated (red) genes are in the top corners. (**F**) Transcript coverage profiles of normalized reads across the longest transcript isoform per gene plotted from 5' to 3' along transcript length divided into percentiles. (**G**) Gene ontology analysis of differentially expressed genes in $APE1_{null1}$ (G) and $APE1_{null2}$ mESC (**H**), showing the top ten enriched molecular function category. (**I**) First panel shows representative immunoblot of APE2 expression of whole cell extracts of mESC clones indicated, Tubulin served as loading control. Second panel shows Ponceau staining of the immunoblot shown in first panel. Third panel: Quantitation of three independent immunoblots, normalized to Tubulin and to wt_1 expression with standard errors shown. (**J**) Sensitivity assays using MMS (J), MMS in combination with the APE1 endonuclease inhibitor CRT0044876 (CRT; **K**), menadione (**L**) or camptothecin (**M**) in wt and $APE1_{null}$ mESC as indicated are shown. Relative absorbance measurements from a metabolic assay (WST-8) and normalized to untreated controls are visualized. Standard error for three independent experiments with technical duplicates are shown. (**N**) Ponceau staining of immunoblot shown in Fig. 1K with mESC lines indicated. Asterisks indicate p-values of unpaired, two-tailed Student's t-test. $*P \leq 0.05$; $**P \leq 0.01$, $***P \leq 0.0001$.*

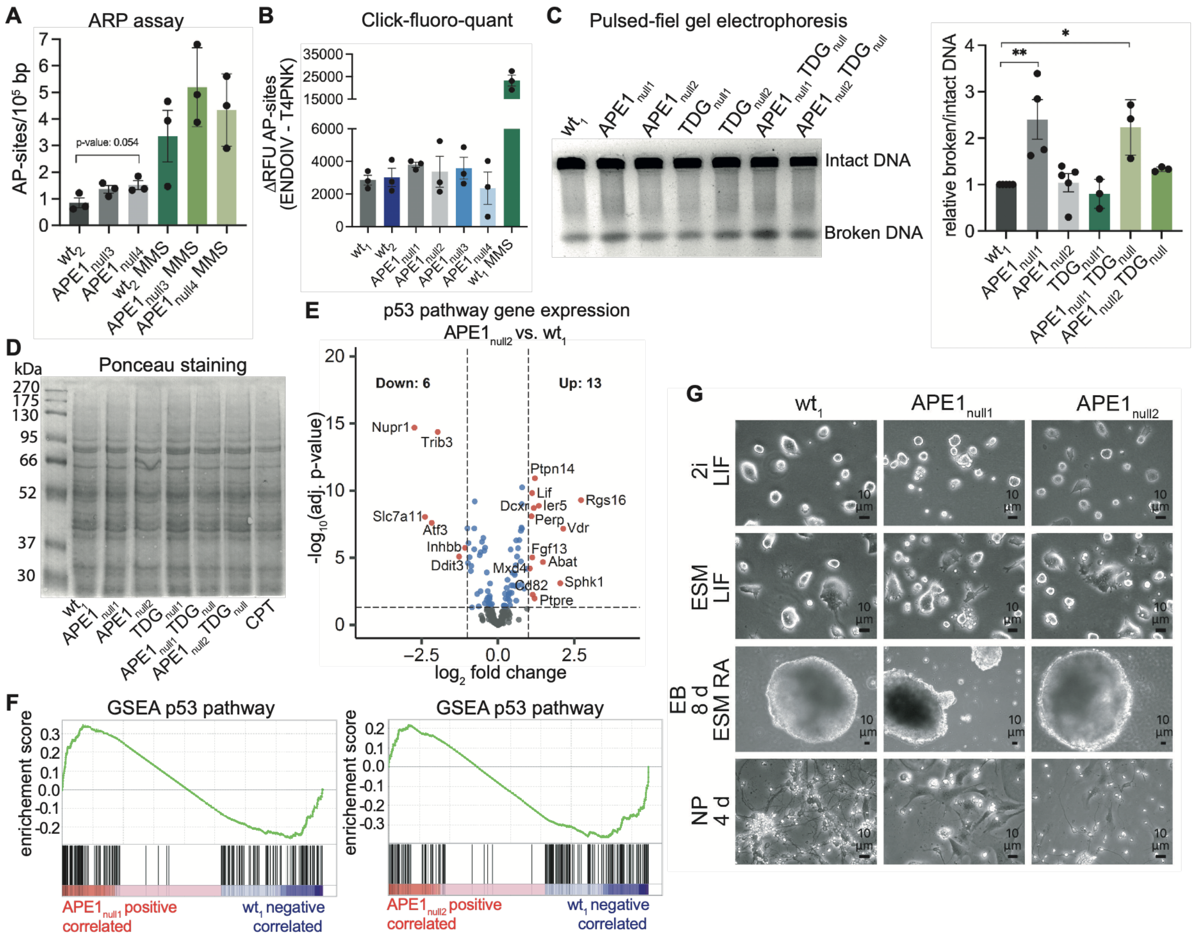

Supplementary Figure 2. Ape1 deletion leaves AP-site levels unchanged and TDG loss does not affect DSB levels
*(**A**) Aldehyde reactive probe (ARP) assay was used to quantify AP-site levels in wt and APE1$_{null}$ mESC in untreated condition as well as with 1h 1 mM MMS treated as indicated. (**B**) AP-site levels were quantified by ligation of a fluorescent marker (click-fluoro quant) and dG-derived AP-sites are reported on the basis of difference in relative fluorescence units (RFU) between ENDOIV and T4 PNK reactions. Shown are means and standard error of three independent measurements per sample. (**C**) Left panel shows representative image of pulse-field gel electrophoresis of mESC lines indicated. Right panel shows quantification of PFGEs, as shown in left panel, with means and standard error of at least three independent experiments. (**D**) Ponceau staining of immune blot shown in Fig. 2C with mESC lines indicated. CPT, campthotecin. (**E**) Gene set enrichment analysis (GSEA) of the p53 pathway (Hallmark gene set) of APE1$_{null1}$ versus wt$_1$ mESC (upper panel) and APE1$_{null2}$ versus wt$_1$ mESC (lower panel). GSEA plot shows enrichment score (green line) reflecting the degree to which genes from the p53 pathway (black) are presented at the top (left, red) or bottom (right, blue) of the ranked list of all genes. Black vertical lines indicate the position of p53 pathway genes within the ranked dataset. (**F**) Volcano plot of differentially expressed genes from the GSEA Hallmark p53 pathway in APE1$_{null2}$ mESC versus wt$_1$ mESC. Significantly (log2FC > ±1, FDR < 0.05) downregulated and upregulated genes (red) are shown and total numbers are depicted in the top corners. (**G**) Representative bright field images of the neuronal progenitor differentiation assay at different stages. d, day; LIF, Leukemia inhibitory factor; EB, embryoid bodies; NP, neuronal progenitor; ESM, embryonic stem cell medium including 15% fetal bovine serum; RA, all trans-retinoic acid. Asterisks indicate p-values of unpaired, two-tailed Student's t-test. *P ≤ 0.05; **P ≤ 0.01, ***P ≤ 0.0001.*

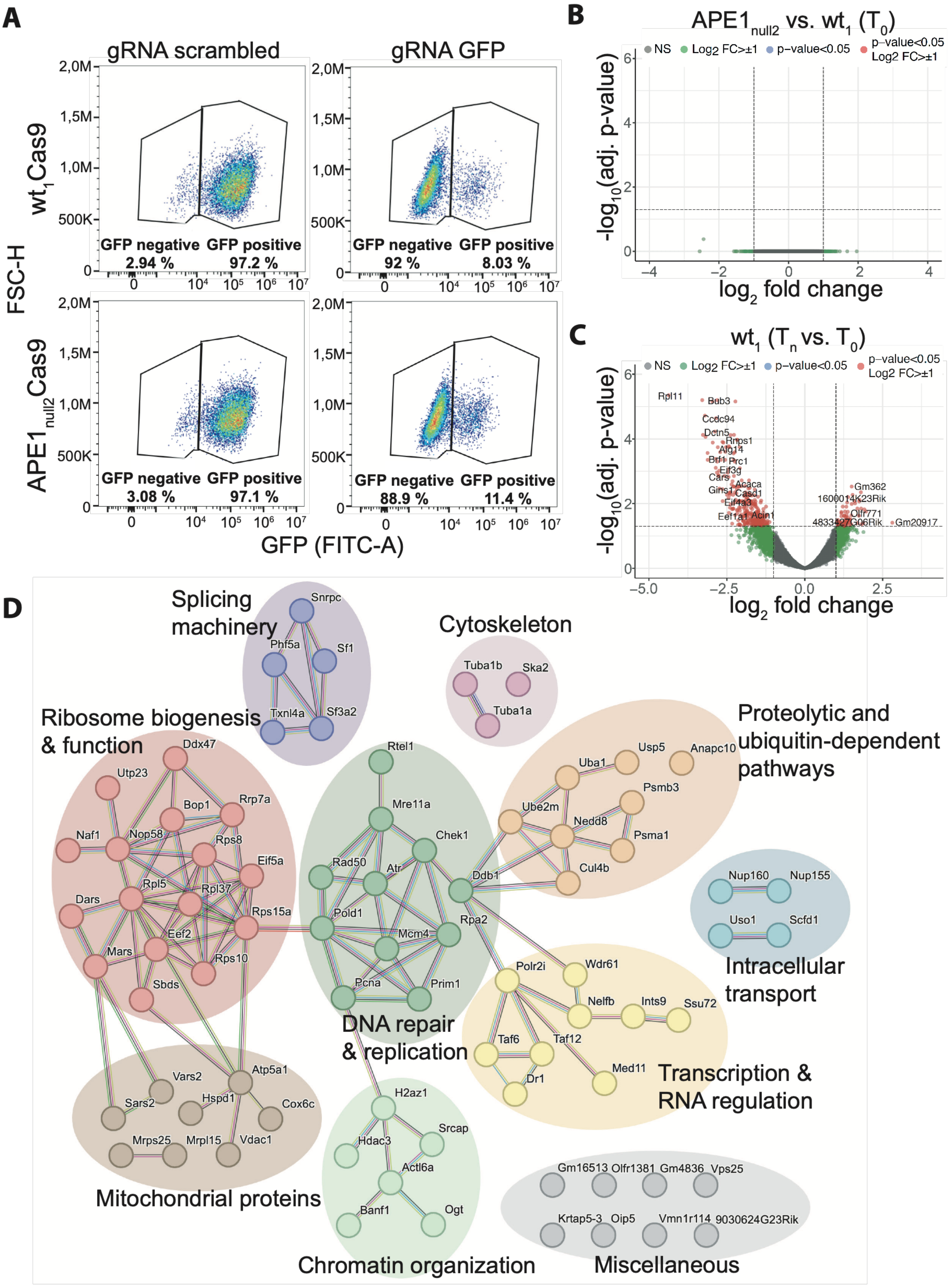

Supplementary Figure 3. Validation of Cas9 activity and genome-wide CRISPR screen hits in and wt and APE1$_{null}$ mESCs. *(**A**) Validation of Cas9 activity by GFP targeting in wt$_1$Cas9 and APE1$_{null2}$Cas9 mESC lines, using flow cytometry. mESCs were transduced with either a gRNA targeting GFP or a scrambled gRNA as control. GFP-negative (left) and GFP-positive (right) populations are indicated within the gated rectangles three days post-infection. FSC-H, forward scatter height (**B**) Volcano plot visualizing distribution of CRISPR gene hits between genotypes (wt$_1$Cas9 and APE1$_{null2}$Cas9) at timepoint T0 (**B**), and between timepoint T_0 and T_{end} within wt$_1$ mESC line (**C**). Significant hits (log2FC > ±1, FDR < 0.05) are shown in red, green labelled genes have a significant fold change (log2FC > ±1) but a non-significant FDR> 0.05. (**D**) Classification of APE1-specific hits into functional clusters using STRING. The figure only shows representative genes for each cluster.*

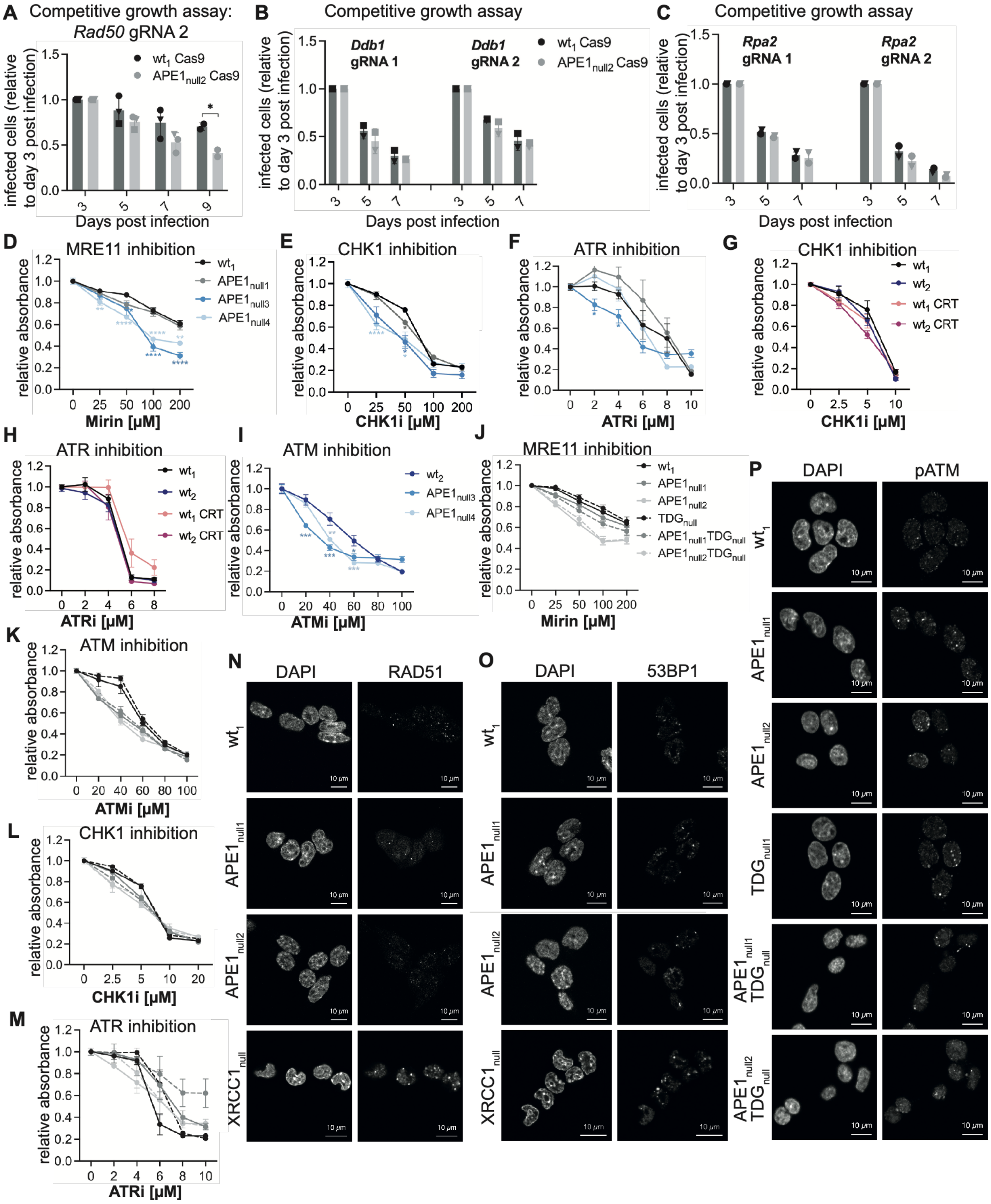
A
Competitive growth assay: Rad50 gRNA 2
infected cells (relative to day 3 post infection)
wt1 Cas9
APE1null2 Cas9
Days post infection
B
Competitive growth assay
Ddb1 gRNA 1
Ddb1 gRNA 2
wt1 Cas9
APE1null2 Cas9
Days post infection
C
Competitive growth assay
Rpa2 gRNA 1
Rpa2 gRNA 2
Days post infection
D
MRE11 inhibition
relative absorbance
wt1
APE1null1
APE1null3
APE1null4
Mirin [μM]
E
CHK1 inhibition
CHK1i [μM]
F
ATR inhibition
ATRi [μM]
G
CHK1 inhibition
wt1
wt2
wt1 CRT
wt2 CRT
CHK1i [μM]
H
ATR inhibition
ATRi [μM]
I
ATM inhibition
wt2
APE1null3
APE1null4
ATMi [μM]
J
MRE11 inhibition
wt1
APE1null1
APE1null2
TDGnull
APE1null1TDGnull
APE1null2TDGnull
Mirin [μM]
K
ATM inhibition
ATMi [μM]
L
CHK1 inhibition
CHK1i [μM]
M
ATR inhibition
ATRi [μM]
N
DAPI
RAD51
wt1
APE1null1
APE1null2
XRCC1null
O
DAPI
53BP1
wt1
APE1null1
APE1null2
XRCC1null
P
DAPI
pATM
wt1
APE1null1
APE1null2
TDGnull1
APE1null1 TDGnull
APE1null2 TDGnull
10 μm

Supplementary Figure 4. Sensitivity of APE1$_{null}$ mESC to the inhibition of different DNA repair proteins.

*(**A**) Competitive growth assay using gRNA 2 against Rad50 to validate CRISPR screen results. Shown are the cell counts of wt or APE1$_{null}$ mESC over time, normalized to scrambled gRNA infection and to the starting timepoint (day three post infection). Different symbols were used to indicate individual experiments. Shown are means of three independent experiments with standard errors. (**B**) Results of the flow cytometry-based competitive growth assay for validation of APE1-specific hits. wt$_1$Cas9 or APE1$_{null2}$Cas9 mESC lines were transduced with two different gRNAs against Ddb1 (**B**) or Rpa2 (**C**). Different symbols were used to indicate individual experiments. Shown are data from two independent experiments with standard errors. (**D**) MRE11 inhibitor mirin (**D, J**), CHK1 inhibitor (**E, L**), ATR inhibitor (**F, M**) and ATM inhibitor (**I, K**) were used at concentrations indicated in a metabolic activity assay comparing wt and APE1$_{null}$ mESC lines. In addition, alone and combined treatments with the APE1 endonuclease inhibitor CRT0044876 (CRT) were performed for CHK1i (**G**) and ATRi (**H**) in wt mESC, using a constant CRT concentration of 200 µM with 3 h pre-incubation. Shown are the relative absorbance measurements, normalized to untreated (0µM) controls. The standard error is shown for at least three independent experiments with technical duplicates or triplicates. (**N**) Representative images of immunofluorescence analyses. Images were taken with a confocal microscope to detect RAD51 (**N**), 53BP1 (**O**), and phospho-ATM (pATM, Ser1937) (**P**) foci in mESC lines indicated. Nuclei are visualized using DAPI. Asterisks indicate p-values of* unpaired, two-tailed *Student's t-test. *P ≤ 0.05. **P ≤ 0.01, ***P ≤ 0.0001*
